# Molecular sampling at logarithmic rates for next-generation sequencing

**DOI:** 10.1371/journal.pcbi.1007537

**Published:** 2019-12-12

**Authors:** Caroline Horn, Julia Salzman

**Affiliations:** 1 Department of Biochemistry, Stanford University, Stanford, California, United States of America; 2 Department of Biomedical Data Science, Stanford University, Stanford, California, United States of America; Temple University, UNITED STATES

## Abstract

Next-generation sequencing is a cutting edge technology, but to quantify a dynamic range of abundances for different RNA or DNA species requires increasing sampling depth to levels that can be prohibitively expensive due to physical limits on molecular throughput of sequencers. To overcome this problem, we introduce a new general sampling theory which uses biophysical principles to functionally encode the abundance of a species before sampling, SeQUential depletIon and enriCHment (SQUICH). In theory and simulation, SQUICH enables sampling at a logarithmic rate to achieve the same precision as attained with conventional sequencing. A simple proof of principle experimental implementation of SQUICH in a controlled complex system of ~262,000 oligonucleotides already reduces sequencing depth by a factor of 10. SQUICH lays the groundwork for a general solution to a fundamental problem in molecular sampling and enables a new generation of efficient, precise molecular measurement at logarithmic or better sampling depth.

## Author summary

Next-generation sequencing enables measurement of chemical and biological signals at high throughput and falling cost. Conventional sequencing uses a process called simple random sampling which requires increasing the number of samples to be able to detect a signal precisely. We have developed a new way to sample, by first performing computations with DNA and then only sampling the output of the computations, requiring a much smaller number of samples to estimate at the same precision as without this method. In common applications such as RNA sequencing or biomarker detection, the method requires 100–1000 fold less sampling, and so reduces cost by 100–1000 fold. This means that the scale and precision of molecular measurement can be dramatically increased, enabling new efficiency in detecting biological molecules.

## Introduction

Deep sequencing has transformed biology and medicine, but remains costly. Depending on the application, billions or more sequencing reads are required to detect rare species in a diverse population; to quantify species, such as RNA transcripts which span a large dynamic range, can require 1–100 million reads. The associated cost of sequencing can be prohibitive for many labs, and scales at a rate that makes sequencing millions of single cells impossible.

There is an underlying statistical reason that such cost is required with current sequencing protocols: they rely on simple random sampling with replacement (SRS), essentially quantifying species through repeated sampling of an urn. Formulated as in classical statistics, the problem is to estimate the multiplicities *n*_*i*_ of N elements *s*_*i*_ in a so-called multiset, and is traditionally solved with SRS: repeatedly sampling elements at random to estimate their abundance.

In many applications, SRS at a depth comparable to current or projected sequencing throughput is insufficient for addressing critical problems in medicine (e.g. next-generation biomarker detection), chemistry (e.g. high throughput compound screens) and biology (e.g. single-cell RNA sequencing); this current approach is insufficient because SRS suffers from intrinsic limitations when: (i) the cardinality of the multiset is comparable or large compared to the number of measurements taken; (ii) large discrepancies exist between the *n*_*i*_ (e.g. expression levels of different transcripts); or (iii) when precise detection of small changes between the *n*_*i*_ is required. A variety of molecular technologies have attempted to address inefficiencies, including targeted or semi-unbiased enrichment or depletion of a population of molecules [1,2]. However, these technologies are only semi-quantitative as they compromise quantification of a set of sequences subject to the depletion and require the depleted or enriched sequences to be prespecified. Similarly, qPCR in principle could be used to quantify a set of pre-known species, but has limited potential to be multiplexed.

Inefficiencies in molecular sampling can be formalized by computing the Shannon information of the sampling distribution; the information of the population distribution in many sequencing experiments is low. We sought to design an efficient molecular measurement platform with theoretically tractable principles that increased the Shannon information of the sampling distribution and that could be realized in experiment. This led us to develop a new general paradigm for molecular sampling that uses computations performed by molecular ensembles to encode the abundance of each species in a sample before measurement, which we call SeQUential depletIon and enriCHment (SQUICH). This paradigm predicts that one can enable experiment-specific sampling designs that only require log or sub-log scale sampling depth compared to SRS, while achieving the same measurement precision.

All approaches that estimate the relative population of molecules in a tube are intrinsically statistical: sampling combined with a statistical procedure gives an estimate of the proportions of molecules observed. In SRS, this estimate is typically the intuitive estimate, which is the empirical proportion of each species observed. In SQUICH, the statistical estimator is a more complicated function of the observed data (see Methods). Some additional experiments are performed for SQUICH; however, sequencing costs still comprise the vast majority of the costs when using SQUICH. Therefore, since next generation sequencing costs are dictated by the total number of molecules that are sampled, sampling depth is the determining factor in evaluating different methods for estimating molecular populations. Accordingly, in all comparisons of how accurately SQUICH and SRS measure the underlying abundance of molecules, we compare the precision of the estimate provided by SQUICH versus SRS on an identical number of samples.

## Methods

**Fig 1:**
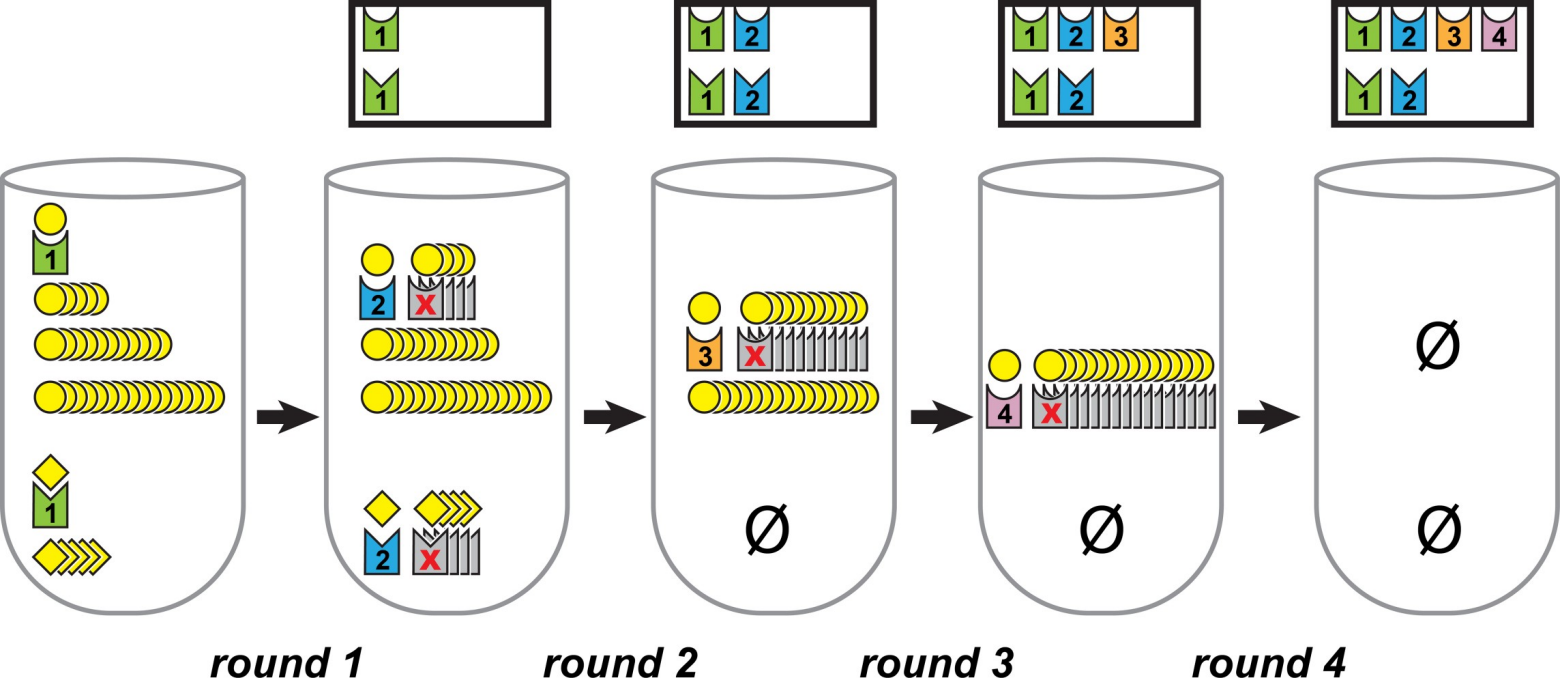
Stylized illustration of SQUICH acting on a sampling tube of shapes, as in Example 1. To the left of the label for each round, we show the tube with the quantities of each shape in the tube before that round. The numbered tags that get attached to each of the shapes in that round are displayed in the tube; the gray shapes with an “X” that look like tags identify the shapes captured and destroyed during the round. For example, the 2nd tube from left represents the contents of the tube after round 1 and before round 2; one of each shape will be tagged with a “2” and be moved to the sampling box during the second round, and some of both shapes (8 for this round) will be captured and destroyed. Above the tube, we see the contents of the box, as it appears before the next round; the box contains the labels for the spheres in the top row and the labels for the cubes in the bottom row. For example, just before round 2, there is one sphere and one cube in the sampling box, each tagged with a “1”. By the end of sampling, if enough samples are taken from the sampling tube, and no observations of “5” for this shape occur, observation of a tag with the number “4” implies that the original number of molecules in the tube exceeds *10*^*3*^ and is at most *10*^*4*^. The total number of shapes in the sampling box is low, requiring very few samples to exhaustively sample it. Note that this example gives an oversimplified version of the method; in particular, the full method enables estimation not only of the order of magnitude, but also of first significant figures (see Example 1.1 and elsewhere in S1 File).

To illustrate the theory of SQUICH, we start with a simple stylized example that demonstrates only a special case of the method’s usefulness (Example 1, illustrated in Fig 1). This example is overly simplified, for the purpose of illustrating the method. In simulation and in the real molecular biology experiments we performed, other sources of error are present. Despite these errors, SQUICH still achieves significant gains in efficiency compared to traditional sequencing, as we will see via these simulations and experiments.

Example 1. Consider a tube containing cubes and spheres, whose quantities an experimenter wants to estimate but does not know; there are in fact 1000 spheres and 10 cubes in the tube. Suppose that there is a physical process that for any number *n* allows up to *n* shapes of each type, but no more than *n*, to be drawn from the tube. Also assume that there is a process to attach numbered tags to each shape. SQUICH consists of repeated rounds of a process of drawing and tagging shapes, which, for the stylized example only, we demonstrate here and in Fig 1.

Round 1: Protocol: Up to 1 of each shape is captured from the tube, tagged with a “1”, and placed into a separate container, which we call the sampling box. Outcome: One object of each shape (cube and sphere) is captured from the tube, tagged with a “1”, and added to the sampling box.

Round 2: Protocol: Up to 1 of each shape is captured from the tube, tagged with a “2” and added to the sampling box. Also, up to 8 of each shape are captured and destroyed. Outcome: 1 sphere and one cube are each captured and tagged and added to the sampling box, while 8 of each shape are captured and destroyed.

Round 3. Protocol: Up to 1 of each shape is captured from the tube, tagged with a “3” and added to the sampling box. Also, up to 89 of each shape are captured and destroyed. (The number 89 is chosen so that at the end of round 3, 100 spheres have been removed from the tube.) Outcome: No cubes are captured, 1 sphere tagged with a “3” goes in the box, and 89 spheres are destroyed.

Round 4. Protocol: Up to 1 of each shape is captured from the tube, tagged with a “4” and added to the sampling box. Also, up to 899 of each shape are captured and destroyed. Outcome: No cubes are captured, 1 sphere tagged with a “4” goes in the box, and 899 spheres are destroyed.

The numbers were chosen to sum to 1000, and thus all cubes and spheres have now been removed. So we do not describe Round 5. Now, the experimenter looks at all shapes in the sampling box. As there is a sphere tagged with a “4”, we know that there must have originally been more than 100 spheres- if not, they would have all been removed from the tube before round 4. If the original number of spheres was more than 1000, there would be at least one sphere left in round 5 to be tagged. We can conclude that the original number of spheres is between 101 and 1000, inclusive. Similarly, as there is a cube tagged with a “2” but no cube tagged with a “3”, we can estimate that the original number of cubes is at least 2, but at most 10.

So, the order of magnitude of each shape in the original tube can be estimated by sampling the 6 objects in the box; on the other hand, SRS requires on the order of *10*^*3*^ samples to make the same inference.

This is an overly simplified example; SQUICH is much more general. For example, the same procedure could take place when starting with, e.g., *10*^*15*^ spheres, allowing a much larger savings in sampling and demonstrating the intuition for why SQUICH enables logarithmic sampling depth compared to SRS. Further, allowing up to 10 cubes or sphere to be tagged in each round allows for estimation of the order of magnitude as above but also estimation of the first significant figure (e.g., the 3 in 3.1 x 10^5^), as we demonstrate with an example in Section 1 of S1 File. In addition, the number of molecules that are captured and destroyed in each round naturally can be varied in different ways: in Example 1, we chose them to grow by roughly a factor of 10 merely for simplicity. The number of rounds is set by the order of magnitude below which precision is desired: e.g., stopping at round 15 means that species with abundance larger than *10*^*15*^ can only be estimated as having abundance larger than *10*^*15*^.

Informally, three properties enable sampling reductions by SQUICH in Example 1: (1) tagging and removal operating independently on each shape; (2) limiting the number of each shape that is tagged and depleted in each round; (3) sampling only tagged shapes. The identities of the species (which in the above example are the shapes) to be sampled must be known a priori: this number could be very large. In our experiments in this paper, the number exceeds 250,000. The abundance of each species is unknown and can be arbitrary: quantification by SQUICH replaces quantification by other sampling approaches.

In practice, the critical properties (1–3) above are fulfilled with a certain configuration of nucleic acids. The place of each shape in Example 1 is taken by a unique oligonucleotide, which we call a target. For each target, sets of certain DNA oligonucleotides called encoders and competitors, each of which hybridize with the target, are the key to SQUICH. An encoder of a particular target has four critical regions: (1) a region of reverse complementarity to the target; (2) a DNA sequence representing the round in which the encoder was added to the original tube; (3) a PCR handle that allows sampling of only targets that *extend on* encoders (i.e. targets that hybridize with encoders and copy a portion of the sequence of the corresponding encoder onto the 3' end of the target molecules by polymerization); and (4) a sequence that, when templating polymerization, causes the extending DNA to terminate, called a terminator. A competitor for a particular target has (1) above, i.e. the same region of reverse complementarity as the encoder, as well as (4). In each round, targets are hybridized with competitors and encoders, and after hybridization, copy through extension on competitors and encoders. For competitors, this extension is the analogue of capturing and removing the shapes in Example 1 because competitors lack a sequence which will serve later as a PCR handle; for encoders, which contain the handle, this is the analogue of tagging and pulling shapes into the sampling box in Example 1 (in which case the target is said to be ‘‘coded”). In addition, the process of targets extending on competitors and encoders entails copying a sequence called a terminator, the only part of the sequence on an encoder or competitor that contains the base “G”; because dideoxy CTP is used during polymerization, the presence of the base G results in terminating the free 3’ end of the target that has extended on an encoder or competitor (see Section 2.2 of S1 File).

As in Example 1, competitors and encoders are added in limiting amounts at each step so that removal and/or tagging of no more than some chosen number of each sequence type can occur in each step. To ensure that only coded molecules are sampled, PCR is used to selectively sample molecules that are targets AND have extended on encoders (Section 2.2 of S1 File). As an aside, if information about competitors were of interest, then competitors could be designed so that targets extending on them could be later retrieved. If targets are in excess of encoders and competitors, the number of targets that extend is limited by the available encoders and competitors. When encoders and competitors are in excess of targets, they compete for binding, which enables the estimation of the first significant figures in scientific notation that was mentioned above. In addition, the abundance of each competitor and encoder can vary by target as may be desired in certain applications; for example, if an experimenter seeks to measure only one species to determine if there are more than *10*^*4*^ copies, then *10*^*4*^ competitors for that species could be added in the first round.

SQUICH is simple to embody in experiment and provably enables logarithmic or even sub-logarithmic sampling compared to SRS for precision desired in ubiquitous sequencing applications, and SQUICH includes estimation of significant figures. For the sake of accessibility, we give a formal theoretical result in only one setting:

**Claim** (Logarithmic sampling): Suppose the abundance of two species are respectively x_*1*_*10*^y*1*^ and x_*2*_*10*^*y2*^ with non-negative integers y_*1*_ *<* y_*2*_, and x_*1*_, x_*2*_ ∈ {1,2, …,9}, and *0 <* p *< 1* fixed. Under certain conditions (as stated more precisely in the Claim in Section 7.2 of S1 File), there is a SQUICH procedure such that ((y_*2*_ +1)/y_*1*_)*log(1/p)* samples suffice to achieve a probability of detection of at least 1-p; SRS requires at least *(10*^*y2-y1-1*^*)log(1/p)* samples. This implies the sampling depth required by SQUICH is logarithmic compared to SRS. Section 7.2 of S1 File contains the proof of the Claim.

The logic of the proof of the claim actually shows how SQUICH can even achieve more general sampling reductions such as sub-logarithmic rates with super-geometric increases in the number of competitors per round.

## Results

Simulation tests of SQUICH performance in real, biologically important applications, rather than as in the stylized Example 1 above, are given in three common application regimes: (1) detection of rare species in the presence of a background of an abundant species (“needle in a haystack”); (2) small fold changes for a small subset of species, within a complex population of a large number of species; (3) quantification of each species in a population with high dynamic ranges, i.e., a population consisting of many species whose abundances vary widely over a large range.

General DNA sequences can be tagged with a user-designed set of known DNA sequences; thus the general measurement problem can be reduced to measurement of sets of DNA sequences of a particular form. To conservatively model SQUICH performance in simulation, for any number n which will determine the sequence length of a DNA oligonucleotide target, we introduce a set of engineered DNA sequences consisting of all molecules matching the format A[(C/G)A)^n^], i.e. all molecules that consists of first one A, then n strings of length 2, each consisting of first either a C or G and then an A; we call these **CGA sequences**. (See Section 2.1 of S1 File for some additional details of the definition.) These CGA sequences are the targets that are the analogues of the shapes in Example 1. Competitors and encoders for a CGA sequence consist of the reverse complement of the sequence, together with auxiliary sequences that identify them as competitors or encoders (as described above and in Section 2.2 of S1 File).

Equilibrium thermodynamics of CGA sequences are modeled in simulations to include inefficiencies and mismatches in oligonucleotide hybridization when the minimum edit distance between targets is one ([3,4] and Sections 2.5 and 3.2 of S1 File). SQUICH can perform more favorably than in our simulation when targets have minimum edit distance of four or more, a design achieved with sphere packing theory [5], or when targets are otherwise designed to have more favorable molecular dynamic properties. In other words, CGA sequences are a convenient way to explain, model and experimentally embody SQUICH, but SQUICH performance is actually optimized by a different design of targets. Experiments in this paper were performed with oligonucleotides containing degenerate bases very similar to CGA sequences (Section 4 of S1 File).

For SRS, total counts for each target sequence were used as the point estimate of abundance, as is standard. For SQUICH, because each observation of a target sequence contains information about both the target and the round the target extended on an encoder, estimation of the abundance of each species in the original pool is a function of the counts observed for each species in each round. Note that in Example 1, the estimation of the abundance of species is trivial. However, in a simulation or real experiment where experimental noise is introduced, such as the molecular dynamics described above, and when the species counts per round are observed through a process of sampling, it is non-trivial to estimate the original abundance. We developed a statistical estimator called the SEM estimator (described in detail in Section 3.5 of S1 File) to convert the measurements of the number of each sampled target sequence, stratified by round, to a point estimate of its total abundance.

**Fig 2:**
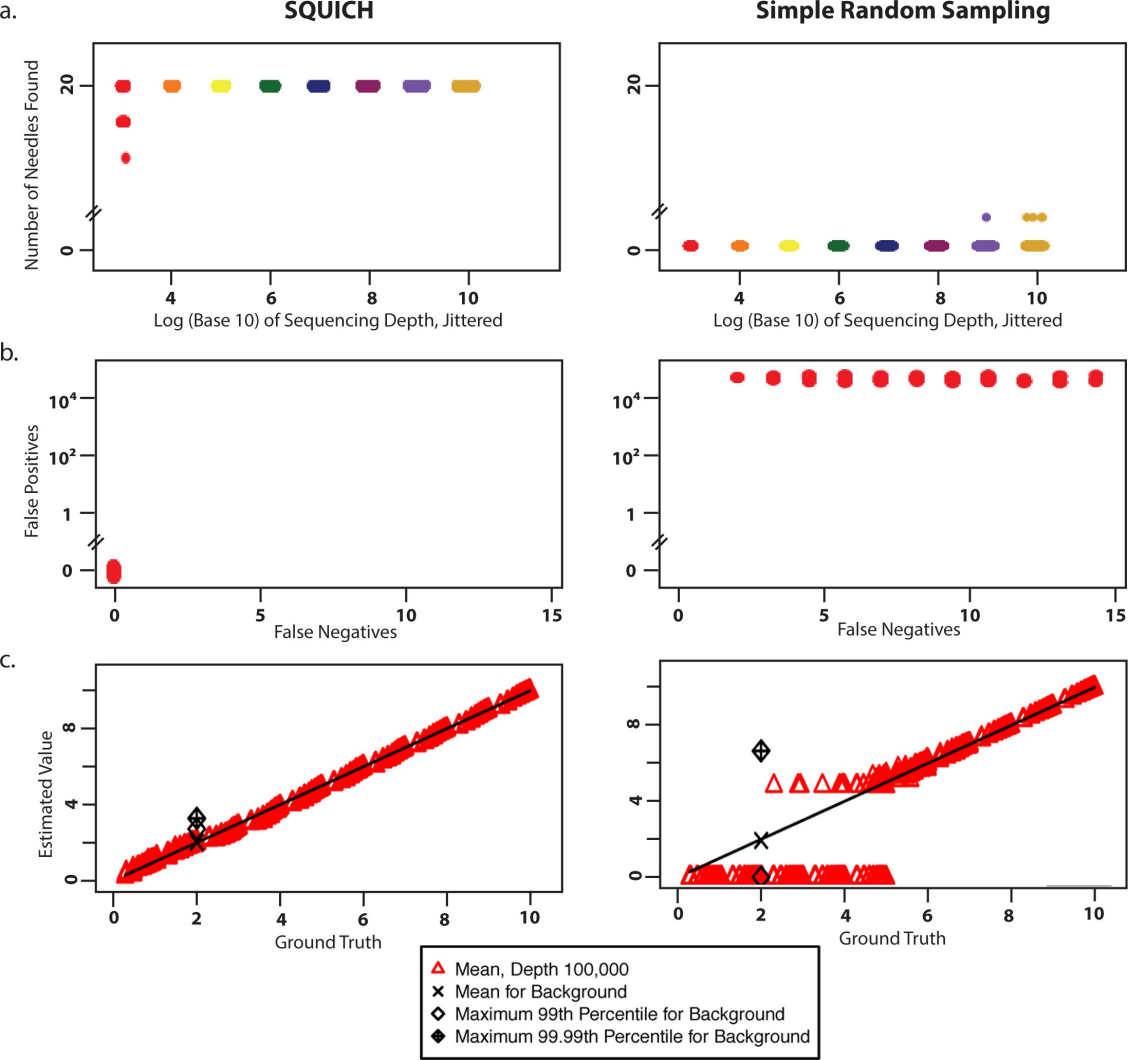
Performance of Simulations. **2a.** Simulation 1: In a background of one species at abundance *10*^*15*^, we compare detection of the “needle” species by SQUICH and SRS for varying sampling depths; each sampling depth is depicted in a unique color, with one point for each replicate. (1000 replicates, x-axis is log_*10*_-scale and jittered). SQUICH (L); SRS (R). Note that due to the number of replicates, there is a large overlap of the points, despite the jittering. **2b:** Simulation 2: SQUICH enables detection of small fold changes, here 2-fold in 20 species, in a background of *>260*,*000* species. Here SQUICH (L) and SRS (R) both use *10*^*5*^ samples. (100 replicates; x- and y-values jittered, y-axis is log_*10*_-scale). **2c:** Simulation 3: Detection performance of SQUICH (L) and SRS (R) (100 replicates each) in quantifying species across 10 orders of magnitude, at a sampling depth of 100,000. SQUICH (L) enables detection of small and large abundances across this dynamic range. (The x-axis and y-axis are log_*10*_-scale and jittered; the axes are labeled by the logs of the values).

Simulation 1 models the “needle in haystack” problem with one species at abundance 10^15^, and *20* “needle” species at abundance *100*. For both SQUICH and SRS, 1000 replicates are performed. SQUICH robustly identifies all needles across all replicates with 10,000 samples (Fig 2A). SRS requires at least 10^9^ samples to detect at least one of the 20 needles (Fig 2A); and it requires 10^15^ samples for the same recall as achieved by SQUICH with 10,000 samples (Fig L of S1 File). For abundance 10^15^, this implies SQUICH reduces sampling depth by a factor of 10^11^; see Figures E-L of S1 File and Table E of S1 File for other values of the abundance.

Simulation 2 tests SQUICH performance where a subset of 20 species are 2-fold more abundant than a background of complexity >*260*,*000*, modeling the complexity needed to detect duplication events of > ~10kb with 1x coverage of the human genome, or a 2-fold enrichment in a chemical or high throughput pooled CRISPR screen. We designed a statistical estimator for SQUICH to identify species enriched above background (see Section 3.5 of S1 File for formal definitions and analysis). With this estimator, 10^5^ samples suffice for recovering all of the *20* enriched species with 0 false positives (FP) across 100 replicates (Fig 2B). To achieve a zero FP rate, SRS requires *10*^*8*^ samples (Fig M of S1 File), requiring at least *10*^*3*^-fold higher sampling depth than SQUICH. This simulation demonstrates that a flexible design of competitor abundance that varies over rounds can increase the power of SQUICH. This case also illustrates a key statistical aspect of SQUICH: competitor and encoder abundance can be modulated to tune FP and false negative rates separately, overcoming an intrinsic limitation of SRS where FPs are functionally related to false negatives as a function of sampling depth.

Simulation 3 tests the performance of SQUICH when the distribution of sampled species fills a high dynamic range (x*10*^y^ for x = 1,..,10 and y = 0, …,9), a situation which arises in measurement of proteins, of environmental microbial DNA, and of RNA abundance when mRNA and abundant noncoding RNA are jointly measured (Fig 2C). Because of the large range of the abundances of the species being estimated, we further developed SQUICH estimation using a distinct estimator for analysis in this problem (see Section 3.5 of S1 File). 10 species were assigned an abundance of each value of the range (resulting in 1000 total species); and ~3000 species were set to a background level of 100. SQUICH fails to detect only 428 of more than 5000 species at a sampling depth of 10^5^; SRS fails to detect 3706 species at the same depth. The log MSE (Mean Squared Error, see S2 Table) for SQUICH is lower than SRS at depths up to and including 10^10^. SQUICH performance with 10^6^ samples also exceeds SRS at depths less than 10^11^ (Fig O of S1 File); at this depth for SQUICH, a mean of only 4 molecules drop out of sampling (i.e., are not sampled).

We also simulated sequencing of mRNA of single cells with a smaller dynamic range of transcript abundance: ~4000 transcripts expressed, including ~2500 transcripts at a basal expression which we set to 100 (n = 2587), and 10 transcripts at each value x *10*^y^ for x = 1,..,9 and y = 0, …,4, and 100 additional transcripts at each level 1:10 (see Section 3.4 of S1 File for details). SQUICH with *10*^*5*^ samples has comparable performance to SRS with *10*^*7*^ samples, as seen in Figure P of S1 File, and also as measured by dropout rate (~*2%)* and log MSE (S2 Table); SRS at *10*^*5*^ samples has a dropout rate of roughly 50%, evidence that SQUICH could significantly improve transcript detection and dropout rates in massive throughput single-cell sequencing [6].

In summary, the simulations show that SQUICH exceeds performance of SRS by 100–1000 or more fold in diverse problems, including detection of expression of rare species, small fold changes, and quantifying species at high dynamic ranges.

**Fig 3:**
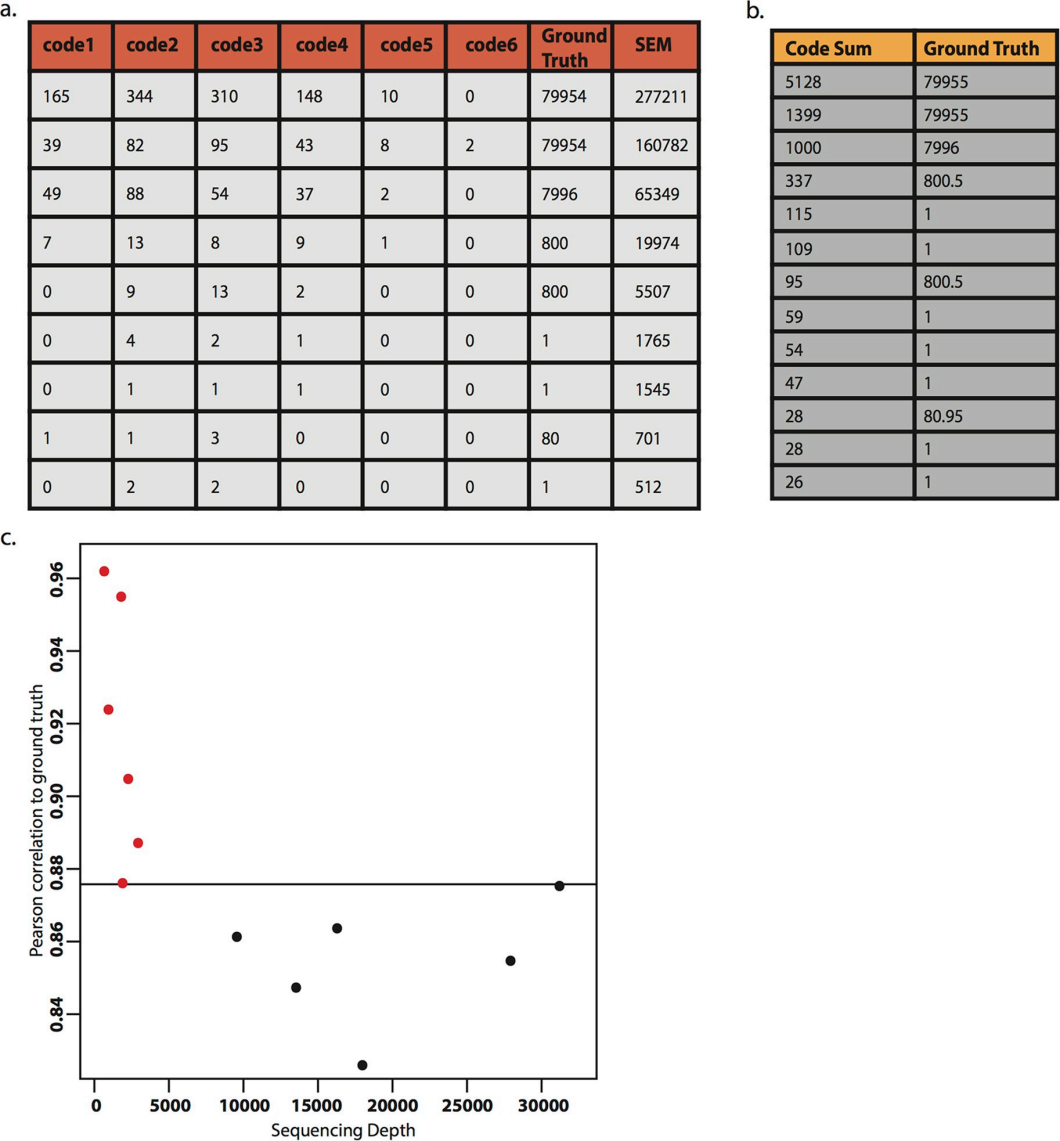
Experimental results. Six SQUICH replicates were sequenced at depths from 1583 to 3305; six conventional sequencing replicates were sequenced to depths of an average of 9 fold greater, from 10345 to 57213 reads. **3a.** Example of sequencing reads in each code round for SQUICH (best representative experiment collapsed over two technical replicates shown) and example computation of statistical estimator SEM. SEM converts SQUICH reads to estimated molecular abundance and is discussed in Section 3.5 of S1 File. **3b.** Left column gives molecules sampled, summed over all rounds; this is the estimate from SRS. Right column is the ground truth. **3c.** Pearson correlation between estimated counts and ground truth (across all species) for each SQUICH replicate (red) exceeded the corresponding correlation for each conventional sequencing replicate (black); this occurred despite the much lower sequencing depths used for SQUICH.

We turn now to a proof of principle experiment to demonstrate that SQUICH works with real molecules. SQUICH, as modeled in simulation, can be directly applied to primary biological samples whenever a screen is conducted with an engineered barcode that is introduced into the sample, e.g. a pooled chemical or genetic screen, with gains in sampling precision illustrated above. To test SQUICH in real next-generation sequencing experiments, we designed a synthetic target library of complexity *2*^*18*^ = 262,144, similar to the set of all CGA sequences with 18 bases that vary, and manually added a set of individual species ranging from *81*x to *80*,*000*x fold over background (Section 4 of S1 File, and Table A of S1 File). SQUICH was carried out with encoder amounts within an order of magnitude of each other in all rounds, and with increases in competitor amounts so that there are 10-fold increases in total molecules (encoders and competitors) in each round (Section 4 of S1 File). To access technical reproducibility, six SQUICH libraries were prepared using encoders that remained constant within an order of magnitude, and also using geometrically increasing concentrations of competitors in base 10. The libraries were sequenced to a mean depth of 2187 reads. Six conventional libraries that model SRS with experimental error introduced during library preparation were sequenced to a mean depth of 19759 reads. Output data is reported in S1 Table. After sequencing, the abundances of all species for both SRS and SQUICH were estimated. For SQUICH, because each read contains information about both the target and the round the target extended on an encoder, we used the SEM estimator mentioned above and described in Section 3.5 of S1 File. (See Fig 3A for an example computation.) In the case of SRS, total counts for each molecule were used as the point estimate of abundance as is standard. The comparison in Fig 3B demonstrates the imprecision of SRS.

In all SQUICH replicates, Pearson and rank correlation between ground truth and estimated abundance exceed the respective correlations for all replicates of conventional libraries, despite SQUICH libraries being sequenced at >9 -fold lower depth (Fig 3; see Section 5, and in particular Figure C, of S1 File for rank correlations); however, the Pearson correlation in one replicate of SQUICH (depth 2407) exceeded the best conventional library (depth 30653) by only ~ 0.00015, but again at a much lower sampling depth than that for SRS.

To control for the high leverage of species with high abundance on correlation values, we used a conservative measure of performance of SQUICH vs. conventional sequencing using a rank based method (Section 5 of S1 File). 5 out of 6 replicates were statistically significantly more sensitive than perfect SRS with no experimental noise (p < .05 in 5 out of 6 replicates; p = 0.138 in one replicate, labeled CH52.03). No p-values were significant for experiments modeling SRS, i.e. for those experiments that lacked competitors. In addition, we formalized a statistical estimate of the empirical depth required by an experimental procedure (SQUICH or SRS) to achieve as accurate an estimate of species’ abundance as would be required to achieve this accuracy with perfect SRS (i.e. without experimental noise including noise introduced during sequencing). This estimate allows us to control for variable sampling depths in SQUICH and conventional libraries. In agreement with the above calculations, we estimate that proof-of-principle SQUICH experiments achieve a 10x reduction in sequencing depth compared to conventional sequencing.

## Discussion

SQUICH is a new theoretical framework for quantifying each of a large number (perhaps millions or more) of species of molecules in a pool, one of the most ubiquitous and important molecular measurement problems today. This theory can be applied to any molecular sampling problem, although here we focus on DNA. Small molecules, proteins and RNA can be tagged with DNA sequences, so common assays and screens all reduce to procedures that SQUICH can improve upon. Moreover, in applications where the sample is limiting, such as biomedical testing, increasing sampling depth is impossible, as sample amplification introduces extra sources of measurement error. The flexibility of the sampling distribution provided by performing molecular computations before sampling expands the scope of statistical algorithms that can be used for estimation. Further, this method provides key advantages when integrated with modern statistical approaches that use assumptions of sparsity to both improve precision in signal detection and reduce resource cost.

For example, SQUICH could be an ideal platform to measure massive single-cell RNA profiles. To illustrate the design of SQUICH for single-cell RNA-Seq, we provide a molecular mapping strategy to combine cell barcodes and gene identity into a single target code as a concise input into SQUICH (Panel b of Fig A of S1 File). Because this strategy involves hybridization, it has a further unique advantage that promises to improve performance in single-cell applications: multiple target codes can be mapped to the same molecule (e.g. RNA) through hybridization with the potential to reduce dropout, resolve isoforms, and overcome 3' bias or the requirement of a poly-A tail.

We predict that SQUICH will allow even further sampling reductions by providing a platform to convert measurement of nucleic acids into target codes that can be measured by approaches such as compressed sensing, which can not be achieved with traditional sequencing [7]. SQUICH enables experiment-specific sampling paradigms that lead to future sampling reductions, for example measuring molecules only when their abundance is above a prespecified value. In proof-of-principle SQUICH experiments, the method achieves 10x reduction in sequencing depth, and we foresee much greater fold reduction by increasing minimum edit distance between sequences in the pool of targets, competitors, and encoders; by increasing purity of oligosynthesis; by improved molecular design; or by experimental designs that enable specific sampling of only species exceeding or depleted by a prespecified fold. This last modification can be achieved with SQUICH by varying the abundance of each competitor (or encoder) by target, so that for example, either encoders in early rounds are omitted, which results in only sampling species exceeding a fixed threshold, or increasing encoders in early rounds and decreasing competitors in order to sample species at low abundance more deeply).

In summary, SQUICH is a new approach for overcoming fundamental limitations in molecular sampling and enables a new generation of efficient, precise biochemical measurement, from screens to detection of rare species in the blood and single-cell sequencing at an unprecedented resolution, with large numbers of potential variations and platforms; moreover, SQUICH lays the groundwork for developing a yet more general methodology that may be able to address multiple fundamental problems in molecular sampling.

## Supporting information

S1 File Supplemental Methods File. (PDF)

S1 Table Information for parsed sequencing data, counts collapsed over each target sequence, each library and each sample. Samples annotated A and B are technical PCR replicates: e.g. A-CH52.01 and B-CH52.01 are PCR replicates and were collapsed for analysis. CH52.01-CH52.06 are SQUICH replicates; CH52.07-CH52.09 are omitted from analysis because they were more favorable to SQUICH than the replicates CH53.01-CH53.06 that were used for analysis.
(CSV)

S2 Table Log loss and drop-out for dynamic range simulations (100 replications) for SQUICH vs. Simple Random Sampling. (PDF)
