# Supplementary material for: Molecular sampling at logarithmic rates for next-generation sequencing
Source: PLoS Comput Biol. 2019 Dec 12;15(12):e1007537. doi: 10.1371/journal.pcbi.1007537 (PMC6932819; doi:10.1371/journal.pcbi.1007537)
Supplement: S1 File — (PDF) [file pcbi.1007537.s001.pdf]

1 Extended Example

Here we present a more complex version of Example 1 from the main text that demonstrates how SQUICH can be used to estimate the first significant figure (e.g. the 3 in 3.1 x 10^6) of an unknown quantity (in addition to estimating the order of magnitude). We hope this also gives the reader more of a taste of the ideas in the method. We do so now in the context of molecules rather than in the context of the shapes of Example 1.

First we describe a little about the process of encoding, the analogue of the "tagging" described in Example 1. Consider the example of a target molecule that is a single strand of DNA. In the encoding process, such a target molecule hybridizes with a so-called encoder molecule and copies a portion of the sequence of the encoder molecule onto the 3' end of the target molecule by polymerization; in short, we say that the target molecule *extends on* the encoder. This tags the target molecule with a unique sequence that, when later observed, indicates that this molecule extended on an encoder in a particular round of the method. Once it does this, the target molecule can no longer extend on any other molecule, as described below in Section 2.2; this then is the analogue of the tagging and capture from the pool in Example 1.

When a target molecule extends on a competitor on the other hand, the target molecule is not tagged, but is simply no longer able to extend on any other molecule, via a similar process as described below; this then is the analogue of the capture from the pool and destruction in Example 1.

In Example 1, some shapes were captured and tagged and some shapes were captured and destroyed. To simplify the exposition, we did not discuss what would happen if there were not enough shapes to go around, so to speak, i.e. if the tagging and destroying processes had to "compete" for shapes. In actual molecular experiments, although this is still a simplification, in each round one indeed introduces encoders (which do the analogue of capturing and tagging) and competitors (which do the analogue of capturing and destroying) at the same time; then, in a sense, these two types of molecules "compete" to hybridize with targets. (In the description of the model and in the Example below, for ease we refer interchangeably to targets (i) hybridizing with, or (ii) extending on, encoders and competitors.) Introducing encoders and competitors at the same time is intentional and is key for estimation of the first significant figure, as we shall demonstrate with this example. We model this competition to hybridize as follows, noting that to simplify the exposition, we omit some important technical aspects of the experiment. (See Section 4 for details of the proof of principle experiments.) Here we have been referring to targets, encoders and competitors only for one species. A key aspect of SQUICH is that the method allows these processes to act independently for each species; thus we continue to focus on one species by itself in the description of the model, which follows:

Model 1:

1. At the beginning of a round, start with T targets (of one particular species).
2. At each round, introduce E encoders and C competitors.
3. (a) If $T \geq E + C$, then all encoders and competitors bind with targets.
   (b) If $T < E + C$, then there are not enough targets to bind with all of the encoders and competitors. We assume that whether or not a particular target molecule extends on either an encoder or competitor occurs with some probability that depends on the relative number of encoders and competitors. So, in the first binding/extending event:

   A target extends on an encoder, with probability $\dfrac{E}{E+C}$;
   Otherwise, the target extends on a competitor.

   After each binding event, the probability of binding would be similar to this last probability, but would depend on the relative proportions of remaining encoders and competitors. In other words, the process is analogous to sampling T objects from an urn containing E encoders and C competitors, but without replacement. Sampling in this setting is described by the hypergeometric distribution.

Observe that in Example 1 in the main text, we did not have any occurrences of condition (3)(b); this was intentional, in order to simplify the exposition.

Now we turn to the example, which demonstrates this model.

Example 1.1

Consider two species of target molecules, A and B, with abundances 1 and 310, respectively, in a sampling tube. An experimenter wishes to estimate the quantities.

For each round i, the protocol consists of introducing some numbers E_{Ai} and E_{Bi} of encoders for A and B, respectively, and some numbers C_{Ai} and C_{Bi} of competitors for A and B, respectively. Recall again that the encoders and competitors for each round are introduced at the same time. The number of encoders and competitors are chosen as follows for A (and likewise for B):

$$\begin{aligned} E_{A1} &= 1 \quad \text{and} \quad C_{A1} = 0 \\ E_{A2} &= 1 \quad \text{and} \quad C_{A2} = 10 \\ E_{Ai} &= 10 \quad \text{and} \quad C_{Ai} = 10^{i-1} \qquad \text{for } i >= 3. \end{aligned}$$

(As in Example 1 in the main text, we note that the total number of molecules up to (and including) the nth round is close to 10^{n-1}, i.e. $\sum_{i=1}^{n}(E_{Ai} + C_{Ai})$ is on the order of 10^{n-1} and similarly for B.)

Now we walk through the rounds of SQUICH for this example and illustrate a potential outcome, bearing in mind that the outcome will have stochastic fluctuations.

Round 1: One encoder for each species, and no competitors, are introduced. One molecule of species A hybridizes with an encoder and extends on the encoder. As noted above, this tags this molecule with a unique sequence that, when later observed, indicates that this molecule hybridized and extended on an encoder in Round 1. A similar process occurs for one molecule of species B, or "1 of B", for short. Thus, 1 molecule of A and B are both tagged and depleted, where by depleted we mean that they are unavailable to extend on any other molecules. (For both A and B, we are in condition (3)(a) of Model 1 above.)

Remaining target molecules that could extend on other molecules: 0 A, 309 B.

Round 2: No A remain. One encoder and 10 competitors for B are introduced. So 1 of B is tagged as being from round 2, and 10 of B extend on competitors. (For B, we are in condition (3)(a) of Model 1 above.)

Remaining: 0 A, 298 B.

Round 3: No A remain. 10 encoders and 100 competitors for B are introduced. So 10 of B are tagged as being from round 3, and 100 of B extend on competitors. (For B, we are in condition (3)(a) of Model 1 above.)

Remaining: 0 A, 188 B.

Round 4: No A remain. There are now 188 molecules of B. 10 encoders and 1000 competitors for B are introduced. So, for B, we are now in condition (3)(b) of Model 1 above, i.e. we have $T < E + C$. Thus any of the 188 molecules of B extend on encoders or competitors with probabilities modeled by the hypergeometric distribution. For the sake of exposition, we think of this in a loose fashion, and so we might expect about

$$\begin{aligned} 188 \times \frac{10}{10 + 1000} &\approx 1.9 \text{ targets to extend on encoders, and} \\ 188 \times \frac{1000}{10 + 1000} &\approx 186 \text{ targets to extend on competitors.} \end{aligned}$$

Of course these numbers are realizations of probabilistic events, and thus could vary widely. But for now, suppose that:

2 targets extend on encoders, and
186 targets extend on competitors.

Remaining: 0 A, 0 B.

Now the process is finished. The experimenter of course determines that there is one molecule of A in the original sample. For molecule B, we know that through round 3, the number that hybridized is the total number of encoders and competitors introduced through that round, namely $(1+1+10)+(0+10+100)=122$. The experimenter will add this to their estimate of the number that hybridized in round 4. This estimate is essentially backed out of what one might expect, but we walk through it. We expect, speaking loosely, that:

$$\begin{aligned}&\text{Realized \# of targets binding with encoders in rnd 4} \approx \\ &\quad (\text{\# of targets in tube before rnd 4}) \times \frac{\text{\# of encoders in rnd 4}}{\text{\# of encoders in rnd 4 } + \text{\# of competitors in rnd 4}}.\end{aligned}$$

The experimenter of course does not know the quantity that they want to estimate, namely the number of targets in the sampling tube before round 4. But the experimenter sees the other quantities in the "approximate equation" above. Thus a good estimate for the quantity to be estimated is found by simply rearranging to obtain:

$$\begin{aligned}&\text{\# of targets in sampling tube before rnd 4} \approx \\ &\quad (\text{Realized \# of targets binding with encoders in rnd 4}) \times \frac{\text{\# of encoders in rnd 4 } + \text{\# of competitors in rnd 4}}{\text{\# of encoders in rnd 4}}.\end{aligned}$$

In this example, we have the estimate

$$\text{\# of targets in sampling tube before rnd 4} \approx 2 \times \frac{10+1000}{10} = 202.$$

Thus the experimenter would estimate that the number of targets B in the original sampling tube was:

$$\begin{aligned}&\approx \text{\# of targets in sampling tube that hybridized in rounds 1, 2 and 3} \\ &\qquad\qquad + \text{\# of targets in sampling tube before rnd 4} \\ &\approx 122 + 202 \\ &\approx 324,\end{aligned}$$

which we see shares the same first significant figure as the ground truth value of 310, and shares the same order of magnitude. Of course SQUICH only produces an estimate of the first significant figure, so it need not give the same value as the first significant figure for the ground truth; certainly it could in theory vary widely, as this is a probabilistic process, just as of course estimates obtained with simple random sampling (SRS) could vary widely.

We emphasize that this example assumes little or no molecular noise (thermodynamic and kinetic fluctuations) that will introduce randomness in the SQUICH procedure when it is implemented with oligonucleotide hybridization, as it is for the proof of principle experiment in this paper. In simulations, we model this thermodynamic noise using conservative parameters taken from the literature on oligonucleotide hybridization (see Section 3.2).

We make one final note: we have essentially assumed here that the experimenter seeks only to measure the abundance of A and B if it is at most 1000; by stopping at round 4, it is not possible to distinguish among abundances larger than 1000.

At the end of this process, sampling from the tagged targets is performed by next generation sequencing; simple molecular biology described below enables only targets that have extended on encoders (not those failing to extend, nor those extending on competitors) to be sequenced.

2 Practical Implementation of SQUICH

In practice, there are many sources of randomness other than the stochastic interaction of a target with an encoder or competitor when using SQUICH experimentally. We discuss some of these here, after first reducing a general problem to a special case that allows us to explain SQUICH more readily.

2.1 Reduction of the Problem

For illustration, consider one problem that SQUICH can be used to address, namely estimating the quantity of a natural DNA or RNA sequence. As this paper is concerned with proof-of-principle, we demonstrate that it suffices to look at the problem of estimating sets of certain oligonucleotides with desirable experimental properties such as low self-complementarity, similar melting temperature, and the potential to be measured with a standardized set of encoders and competitors. We call these oligonucleotides CGA sequences and define them here.

Definition 2.1 (CGA Sequence) *For any positive integer n, a* ***CGA Sequence of order*** $\mathbf{n}$ *is a sequence of length $2n+1$ taking the following form:*

$$[X]\,\underbrace{A[C/G]A[C/G]A\ldots[C/G]A}_{2n+1},$$

where $[C/G]$ denotes a nucleotide that is either C or G. In other words, the portion of the sequence that is of length $2n+1$ is a nucleotide sequence consisting of: (i) an A; then (ii) n strings of length 2, each of which takes the following form: first either C or G, then A. The sequence X denotes a primer binding site at the 5' end of the remainder of the sequence, and X is common to all targets. The set of all 2^n variants of the above is called the ***complete set of CGA Sequences of order*** $\mathbf{n}$.

Here, and throughout, we adhere to the convention of writing sequences from 5' to 3' left to right.

Returning to the practical problem, one wants to estimate the quantity of, say, a particular species of RNA molecule. An example of how to do this is depicted in Figure A. One first creates probes of a certain type, namely with one region that hybridizes to the specific RNA sequence and another following region that consists of a particular CGA sequence. In practice, this will occur for multiple different RNA sequences, and each one will be associated with its own particular CGA sequence, with the length of the CGA sequences chosen to be large enough so that this is possible, which of course depends on the number of different RNA sequences whose quantities one wants to estimate. By designing an affinity tag (e.g. streptavidin) and cleavable tag (e.g. uracil cleaved with the USER enzyme) on the probes (see panel (a) of Fig A), this molecular biology procedure essentially converts each RNA sequence to the corresponding hybridized CGA sequence. In this way, the molecular procedure for SQUICH on endogenous RNA is reduced (in the computer science sense) to measurement of CGA sequences. Thus the original problem is equivalent to the problem of estimating the quantities of the corresponding CGA sequences, a problem that is easier due to the characteristics of CGA sequences: less self-complementarity, similar melting temperature, and the potential to be measured with a standardized set of encoders and competitors.

2.2 SQUICH with CGA Sequences

We now describe the SQUICH process for estimating quantities of CGA sequences in particular. Recall that we use the word target to describe a sequence whose quantity is to be estimated. SQUICH would generally be used with

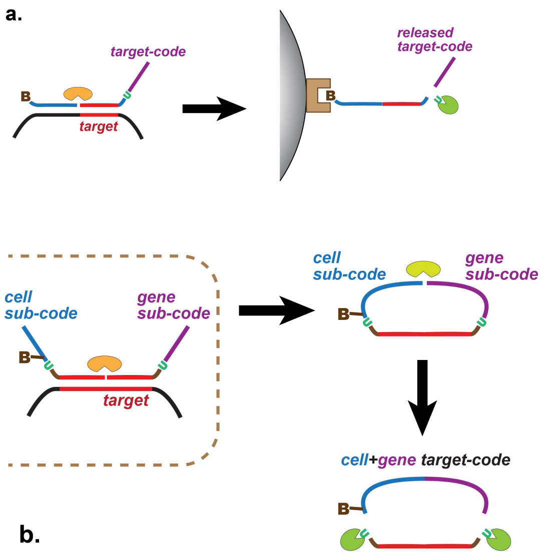

Figure A: Signal conversion with SQUICH procedure, illustrating one of many approaches for converting endogenous nucleic acids to SQUICH target sequences using ligation (depicted as an orange pacman), Biotin (B), Uridine (U), and an enzyme that cleaves U (in green). (a) Ligation of two oligos, one encoding “part1” and one encoding “part2”, is templated by an RNA; one of the oligos is biotinylated at the 5’ end. Together the oligos specify a mapping from endogenous sequence to target code (right side); biotin pull-down and elution release the SQUICH target for input into SQUICH. (b) The same framework is applied, though with each probe containing a “part” of the target code, the cell sub-code and the gene sub-code. After ligation, samples are pooled and circularized, joining the two “parts” to make a complete SQUICH target which is similarly eluted for measurement by SQUICH. After SQUICH target abundance estimation, the target is decoded to assign the abundance of the transcript and its cell origin.

many targets simultaneously, but for illustration, we will look at a single example target throughout, namely

$$\mathcal{S}_0 = [X]AGAGACA,$$

where X is the primer binding site.

Problem: The problem to be addressed is: given a pool of target CGA sequences, estimate the quantity of certain of the CGA sequences. Abstractly, we have a so-called multiset of these sequences, so there may be 10^6 copies of $\mathcal{S}_0$, and 9 copies of another sequence, say, $[X]AGAGAGA$, and many more copies of other sequences. The goal may be, for example, to estimate only the quantity of $\mathcal{S}_0$, or it may be that one wants to estimate the quantities of many (pre-chosen) CGA sequences. In practice, we may of course want to estimate concentrations of sequences, rather than quantities.

Fix a number n_R of rounds. We assume that the experimenter has some knowledge of reasonable upper bounds for the quantities and that this knowledge will allow for a good choice of this number. In each round, one simultaneously introduces some number of encoders and competitors, which we describe here.

An experimenter will associate each round with a particular nucleotide sequence, which we call a **code**.

We now describe the encoder molecules. Note that one can introduce either: (i) a set of encoder molecules with complements matching every possible target sequence, so that the quantity of encoders is the same for every possible target sequence; or (ii) a set of molecules with complements for each particular target sequence, so that the quantity of encoders can vary by target sequence; we detail this more below.

Encoders: *Encoders take the form*

$$t\ [PCR\ handle]\ [code_i]\ \underbrace{T[C/G]T[C/G]T\ldots[C/G]T}_{2n+1},$$

where $[code_i]$ is the code associated with the ith round, $[PCR\ handle]$ is a PCR handle, i.e. a sequence enabling priming in PCR; t is a sequence at the 5' end called a "terminator" (described more below); and the right sequence denotes the set of reverse complements of all possible CGA sequences of order n. The terminator sequence is a sequence that, when templating polymerization, causes the extending DNA to terminate. The terminator is the only part of the sequence containing the base "G"; by using dideoxy CTP during polymerization, any free 3' end that has extended on an encoder– i.e. that has used the molecule as a template to copy using polymerase extension– gets terminated. Together, these components define encoders in the context of sets of CGA sequences, i.e. in the case (i) described before the definition.

On the other hand, for a particular CGA sequence denoted S, corresponding encoders take the form

$$t\ [PCR\ handle]\ [code_i]\ [reverse\ complement\ of\ S\,].$$

In the ith round, the number of encoders introduced is denoted E_i (or E_{ij} if we want to emphasize its dependence on a target sequence indexed by j).

For our example $\mathcal{S}_0$, encoders take the form

$$t\ [\text{PCR handle}]\ [\text{code}_i]\ TGTCTCT.$$

As mentioned in the definition of encoders, we could, for each round, introduce E_i encoders of the form above for every single combination of the variants of G or C– i.e. for all 2^n variants, or in other words, for the complete set of CGA Sequences of order n– with each adjacent to code$_i$ and a PCR handle. In this case, any encoder is perfectly complementary to only one specific target (ignoring, for now, kinetic and thermodynamic noise which can cause each target to hybridize to multiple species). In an alternative case, one might design the set of encoders so that the number of encoders E_i in round i varies by target. As mentioned above, one may actually in practice introduce a certain concentration of encoders.

Competitors: *Competitors take the form*

$$t\ \underbrace{T[C/G]T[C/G]T\ldots[C/G]T}_{2n+1},$$

where t is a sequence at the 5' end called the "terminator". As above, the terminator is a sequence that contains the base "G", which is absent in sequences used for coding and PCR handles. In the extension reaction, we include dideoxy CTP, a nucleotide that, after being incorporated, can not template a growing chain. Thus, after extending on a terminator, a target cannot extend on any other molecule in the pool because it no longer has a free 3' end, i.e., it cannot use the molecule as a template to copy using polymerase extension. When a target molecule binds and extends on the competitor, it will no longer be free to hybridize and extend on an encoder even if the target and competitor are denatured in a future round. In the ith round, the number of competitors introduced is denoted C_i (or C_{ij} if we want to emphasize its dependence on a target sequence indexed by j).

For our example $\mathcal{S}_0$, competitors take the form

$$t\,TGTCTCT.$$

Now we describe the method, which only has a few modifications from the description in Example 1.1.

An experimenter wants to estimate the quantities of some k pre-chosen target CGA sequences $\{S_1, S_2, \ldots, S_k\}$ of interest in a tube; there may be other molecules in the sample as well. The experiment proceeds first in what we call rounds, from round 1 to the last round n_R, and then has additional steps. The rounds take place in step (A) below.

An experimenter may choose to insert known quantities of certain spike-in molecules (i.e. targets of pre-specified abundance, that is, with known ground truth); these help to estimate absolute quantities rather than only relative quantities, as will be seen later. Additionally, targets contain a common sequence at the 5' end serving as a forward priming site which will be significant in step (B) below.

(A) Rounds $i = 1, 2, \ldots, n_R$**:** In round i, for each target CGA sequence S_j of interest, E_{ij} encoders and C_{ij} competitors (for $1 \leq j \leq k$) are placed into the tube. Note that the encoders and competitors may vary for each target sequence; moreover, their quantities could also vary by target sequence. In these rounds, targets hybridize with encoders and with competitors until targets are depleted. Hybridization is performed in the presence of polymerase, so extension on encoders and competitors, and, subsequently, blocking of their 3' ends, occurs in each round.

(B) After Round n_R, PCR is used to amplify the targets which have extended on encoders. This is accomplished by using the forward primer that is only present on targets and a reverse primer only present on encoders. Thus, the only amplified molecules will be targets that have extended on encoders.

(C) The resulting pool is sampled for sequencing. Note that because of the amplification by PCR, sequencing samples only targets that have hybridized and extended on encoders. When sequenced, each target provides information both about the sequence of the target and its abundance, through the code of the encoder. As a result, far fewer samples are required, compared to simple random sampling, to achieve a similar quantitative inference about initial abundances.

(D) These samples are sequenced and counted.

Analysis: The counts of the sequenced targets which extended on encoders are used to construct an estimate of the original quantities for each of the target sequences of interest. We gave an example of such a computation in Example 1.1 and will give details below.

2.3 Illustration of SQUICH with CGA Sequences

Figure B gives a toy example of SQUICH with CGA Sequences.

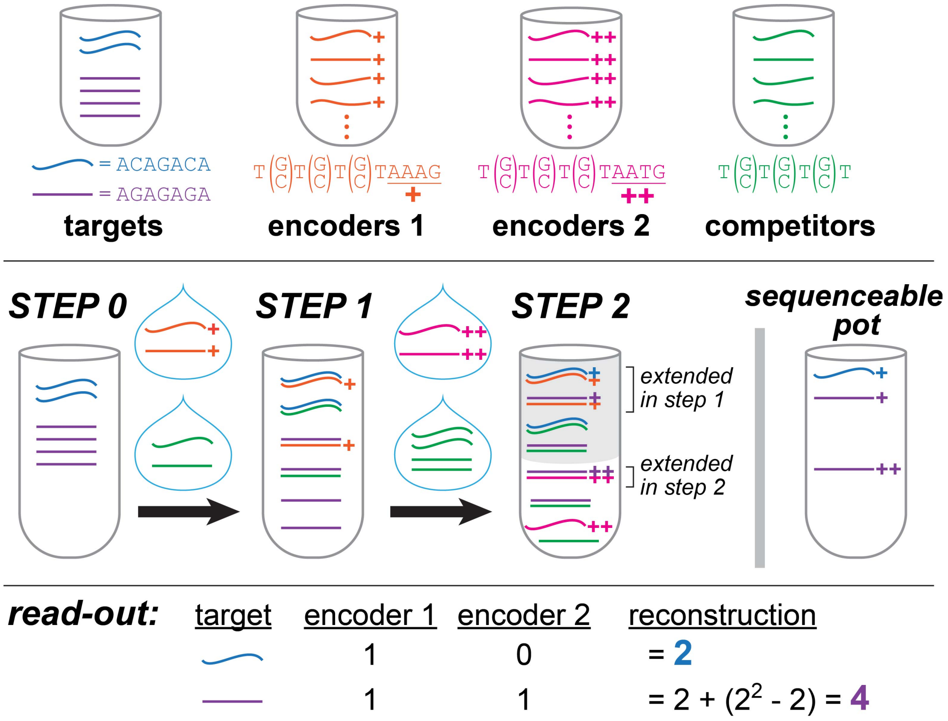

Figure B: The SQUICH components, procedure, and output for a set of CGA sequences. **Row 1** shows the following four components: (i) the target mixture, with two species, ACAGACA (shown as curved) and AGAGAGA (straight) (with the primer binding site not displayed, for simplicity); (ii) encoder round 1 (with code AAAG) and (iii) encoder round 2 (with code AATG) mixtures; and (iv) the competitor mixture. Note that each of the last three mixtures consists of 8 different types of sequences. Some of the encoder and competitor sequences (shown $3' - 5'$ here) contain one region that is complementary to the targets, e.g. the region TGTCTGT for the target ACAGACA. In this example, the encoder oligonucleotides contain a region (not shown) at the $5'$ end that can serve as a template for target extension in the presence of a DNA polymerase. Similarly, the terminator sequences on competitors and encoders are not shown here. **Row 2** shows the addition of encoders and competitor through two rounds of the SQUICH procedure. **Round 1:** the encoder 1 and competitor oligonucleotides are added simultaneously in small, equal amounts to the target mixture. Those targets (curved or straight) that are hybridized to their encoder 1 complements are extended by a DNA polymerase to incorporate the bases complementary to code 1, namely TTTC, into their $3'$ ends. Those targets that are found by the competitor produce a dead end product. **Round 2:** encoder 2 and a two-fold increase of competitors are added simultaneously. Fewer target:encoder 2 extension products (with TTAC) are made because of the larger amount of competitor and/or the decreasing amount of targets. At the end of the encoding rounds, the targets that were extended to include the encoder 1 and encoder 2 sequences can be used as templates for DNA sequencing. Both the target region and the encoding regions are sequenced. **Row 3** shows the DNA sequencing results for this example. One counts all combinations of targets and encoders. The "curved" target appears only with encoder 1, indicating a species of low abundance. The "straight" target appears with both encoder 1 and encoder 2, indicating it is more abundant than the "curved" target. The original abundances of the "curved" and "straight" sequences can be reconstructed to be 2 and 4, respectively, based on the two-fold increase in the amount of competitor added in Step 2. In this simple example of SQUICH, just three DNA sequences can be used to estimate the abundances of a mixture of 6 sequences. Note that we have omitted the PCR and sampling steps for simplicity.

Now we present an example of using SQUICH with CGA sequences in the case of one rare and one abundant target.

Example 2.2. SQUICH with CGA sequences in the case of a rare and abundant target

In this example, the goal is to jointly measure the abundance n_1 of a sequence s_1, which we think of as the "needle", and the abundance n_2 of a sequence s_2 with n_1 much smaller than n_2. We assume that $n_1 \geq C_1$ where C_1 is a fixed constant which can be interpreted as a lower limit of detection. Fix n and assume that s_1 and s_2 are CGA sequences of order n. Suppose SQUICH is performed with the competitor and encoder sets of order n above, with 10^i equaling the total number of encoders and competitors up to the ith round, and suppose that the encoder abundance is constant with $E_i = 10$ for all i. We assume $n_1 = 10$, so in the first round, all molecules of s_1 hybridize and extend on the encoder, but only n_1 of the molecules of s_2 do so. After this round, all extendable targets from the first species have been extended and coded, and will never extend on a higher code in future round. In contrast, the abundant species will be sequentially hybridized and extended on both encoders and competitors until the $r := \lceil \log_{10}(n_2) \rceil$ round when all of the original n_2 molecules have extended. If $n_2 = 10^8$ (and $n_1 = 10$, as stated earlier), the underlying molecular composition after SQUICH would then be:

sequence	code1	code2	code3	code4	code5	code6	code7	code8	code9
$s_1 : n_1 = 10$	10	0	0	0	0	0	0	0	0
$s_2 : n_2 = 10^8$	10	10	10	10	10	10	10	10	0

In a real sequencing experiment, the molecules represented in the table are typically amplified with PCR and then sampled on the sequencer. Thus, the observed counts – reads from each sequence– will be sampled from a Poisson distribution or multinomial distribution when we condition on total number of reads observed. We note that the multinomial and Poisson distributions are close approximations of each other in the sampling regimes used in SQUICH and hence we use them interchangeably. The parameters representing sampling rates are represented by the numbers in the above table. The following table was generated by simulating random variables with the underlying Poisson parameters fixed above.

sequence	code1	code2	code3	code4	code5	code6	code7	code8	code9
$s_1 : n_1 = 10$	3	0	0	0	0	0	0	0	0
$s_2 : n_2 = 10^8$	14	5	15	13	10	7	10	11	0

It is clear that the needle is detected with SQUICH with 100 reads; also, on the order of 10^8 reads are expected to be required to detect this needle with high probability with SRS. This idea is formalized later.

2.4 Estimation of Absolute Counts

Note that in step (C) in Section 2.2, one samples from a pool of molecules, and these molecules have been amplified with PCR. Thus we lose information about the absolute numbers (or concentration) of these molecules, and only retain knowledge about the relative values of the quantities.

To obtain estimates of absolute abundance, we use standards or spike-ins, a common approach in molecular biology. To do this, spike-in molecules of known quantities are introduced, before the rounds of SQUICH. Abundance of spike-ins are measured alongside target sequences, and then their abundance is estimated using the SQUICH procedure. Then one uses the ratio of (i) the actual quantity of spike-ins to (ii) the estimates given by SQUICH for the spike-ins. This ratio can *essentially* be used to inflate or deflate the estimates given by SQUICH for the targets to get an estimate of the absolute quantities of the targets; there are some more details, which will be discussed in Section 3.5. Additionally, spike-ins can be used for a certain method of bracketing estimates as well, as will be discussed in Section 3.5.2.

2.5 Experimental Noise

There are practical aspects that one must take into account when moving from the idealized version of SQUICH to experiment. We will of course model these in our simulations as well.

1. **Hybridization Inefficiency** Some fraction of the encoders and competitors may fail to hybridize with any of the target sequences under the conditions of an experiment. We term this *encoder failure* and *competitor failure*, respectively. Similarly, some fraction of the targets may fail to hybridize with any encoders or decoders; we term this *target escape*.

2. **Thermodynamic Bias, or Off-Target Hybridization.** Some fraction of the sequences for a particular target may hybridize with an encoder having a substring that is not the exact complement of the target sequence. For example, the target $[X]ACAGACA$, expected to hybridize with the encoder t[PCR handle][code$_i$]$TGTCTGT$, might instead hybridize with the encoder t[PCR handle][code$_i$]$TCTCTGT$, which has a mismatch with the last C of the target.

These practical aspects will indeed be modeled when we run simulations, which we turn to next.

3 Simulation Methods

SQUICH was simulated following Section 2.2 with additional variance introduced by modeling of hybridization inefficiency and thermodynamic effects. After SQUICH coding, each coded species was sampled with replacement and compared to simple random sampling (SRS) directly from the original pool. We first define some vocabulary.

- **Code-extended targets, or encoded targets:** target molecules that have extended on an encoder, i.e. targets that contain a target sequence upstream of a code sequence.
- **Competed targets:** target molecules that have hybridized and extended on a competitor
- **Complementary encoder for a target:** this is the encoder that corresponds to a given target; i.e. it has a subsequence complementary to the target.
- **Complementary competitor for a target:** this is the competitor that corresponds to a given target; i.e. it has a subsequence complementary to the target.

Note that competed and code-extended targets do not have free 3' ends because the last nucleotide incorporated when they polymerize on an encoder or competitor is a dideoxy.

3.1 Modeling of Hybridization Inefficiency

There may be some failure of encoders, competitors and targets to hybridize, for example if there is DNA binding to the tube in an experiment. Inefficiencies are modeled by assuming that in each round, molecules failing to hybridize are removed temporarily from the pool of molecules for the round but then return in the following round. The number assumed to be removed from each round is modeled by simulating a random variable according to a binomial distribution with a fixed probability. In our simulations, the probabilities for each of the three types of failures to hybridize (as described in (1) of Section 2.5) have been set to 0.1, corresponding to reports by Integrated DNA Technologies (IDT) of typical empirical efficiencies of annealing and extension in thermocycling conditions, the molecular procedures performed in SQUICH (www.idt.com). We also ran all of the simulations again with all three probabilities set to 0.4, and they all look very similar to the original results, with the exception of only somewhat similar results for Simulation 2, which we therefore report in Section 9.2.

3.2 Modeling of Thermodynamic Effects

Off-target hybridization effects are modeled as follows. First, we assume a worst case situation for SQUICH: we assume that targets, encoders and competitors are all oligonucleotides such that for each target (respectively encoder, competitor), at any variable nucleotide in the target (respectively encoder, competitor), there exists another target (respectively competitor, encoder) one mismatch away from it. This situation is less favorable than a superior design that would use sphere packing theory to set each pair of targets at an edit distance larger than 1.

We assume that the relative probability of mismatching between imperfectly complementary oligonucleotides (e.g. a target and an encoder) is a function of a discrimination factor χ and the number j of mismatches in the imperfect complement as follows. For a target t with a complementary encoder e_0 and having j mismatches to (the relevant subsequence of) an encoder e_j, [3] and [4] show that there is an oligonucleotide design (e.g. including auxiliary molecules such as "protectors") for any library, including the one described here, such that

$$\frac{P(\text{t is bound to } e_j)}{P(\text{t is bound to } e_0)} = \frac{1}{\chi^j},$$

and similarly for competitors.

If there are N molecules of a target CGA sequence, and there are n regions of the sequence taking the value G or C, then the above implies that the expected number of targets hybridizing to any encoder or competitor having from 1 up to k mismatches is

$$N \sum_{j=1}^{k} \binom{n}{j} \chi^{-j}. \tag{1}$$

$k = 5$ is used in simulations; the energetic penalty of 4 or more mismatches is very high [A], and 5 allows for further caution. Our simulations use values of χ of 50, noting that conservative estimates of reports in the literature are 100 and 500 ([3],[4]).

Perfect complementarity of targets and encoders is not required to fulfill the conditions for SQUICH. Many of the sub-optimal energetic (i.e. imperfect complement) configurations in SQUICH are indistinguishable from optimal energetic hybridization where all duplexes are perfect. An example of this is as follows. Let X and X' denote the vector of perfectly duplexed targets and encoders during a SQUICH round, and X'_π denote a permutation of the entries in X' such that X can prime on X'_π in each entry. It is clear that for any permutation π, the sequence of X after it primes on X'_π will not depend on π, because the hybridization sequence in the encoder is never copied onto the target; it is only the code in the encoder that is copied onto the target. Finally CGA codes are suboptimal because each has edit distance 1 from n other target codes. Our simulation results could have been improved by considering a set of targets where each pair is 4 or more mismatches away from each other. In such a case, thermodynamics predicts almost no imperfect duplex binding.

3.3 Implementation of Simulations

There are three classes of simulations, involving various choices for the number of distinct species of target molecules and quantities of each, as well as various choices for spike-ins. We discuss the simulation of the SQUICH procedure in this section. The choices made for the parameters just mentioned are described below in Section 3.4. The estimators for these simulations will be discussed in Section 3.7.

We now discuss the simulation of the SQUICH procedure over each round i from $1, \ldots, I$ for some positive I, where we start with N copies of the particular target molecule. Note that generally N is unknown (except in the case of the spike-ins) and that it is the quantity we wish to estimate, but here of course we are modeling what the simulation process will produce. Then we introduce the following molecules in the ith round, for $1 \leq i \leq I$:

$$E_i \text{ encoders}$$
$$C_i \text{ competitors.}$$

These choices are to be made without knowledge of the quantities of the targets (but of course an experimenter might have some knowledge of the class of problem and might choose the values E_i and C_i accordingly, as do we in the simulations). It is worth noting that these values could vary by species, but in our simulations, the values are kept constant across species. Also we define

$$n_i = \text{ Number of target molecules available to hybridize at the beginning of the } i^{th} \text{ round for } 1 \leq i \leq I;$$

observe that $n_1 = N$. One can think of n_i as the number of targets "remaining", although they have not actually been removed from the pool, but rather are just not available to hybridize.

In each round, the modeling proceeds as follows; each is a random variable with a computable but quite complicated distribution. The overall stochastic process, i.e. the vector of these random variables, is a martingale.

1. **Target Escape (Hybridization Inefficiency):** Some number $n_{i,escaped}$ of the targets are assumed to not hybridize in round i; these return to the pool for the next round. The number $n_{i,escaped}$ is drawn from the binomial distribution with probability *p.escape* and n_i trials, as discussed in Section 3.1. Thus in the ith round there are $n_{i,active} = n_i - n_{i,escaped}$ targets that are assumed to completely hybridize. One detail is that if n_i is large, the normal approximation to the binomial is used because of definitions of variable types in the R programming language.

2. **Encoder and Competitor Inflation (Thermodynamic Effects):** Thermodynamic effects are modeled by assuming that the number of potential encoders (respectively, competitors) is equal to the sum of (i) E_i (respectively, C_i) and (ii) the quantity in equation (1), where N in that quantity takes the value E_i (respectively, C_i). We call this number of potential encoders (respectively, competitors) $E_{i,potential}$ (respectively, $C_{i,potential}$).

3. **Encoder Failure (Hybridization Inefficiency):** Beginning with the potential number of encoders $E_{i,potential}$, some number $E_{i,failed}$ of encoders are assumed to fail to bind to targets, which is modeled according to the binomial distribution (or the normal approximation to it) with some probability $p.encoder.failure$, just as with target escape. The remaining $E_{i,active}$ encoders, defined by $E_{i,active} = E_{i,potential} - E_{i,failed}$, are assumed to completely hybridize.

4. **Competitor Failure (Hybridization Inefficiency):** A value $C_{i,active}$ is simulated, exactly by analogy to encoder failure, with some probability $p.competitor.failure$.

5. **Modeling of Hybridization:** There are two cases.

   (a) $n_{i,active} >= E_{i,active} + C_{i,active}$**:** In this case, there are enough targets to bind with (and extend on) all encoders and competitors. Then the resulting number of code-extended targets and competed targets is simply:

   $$\text{Code-Extended Targets for Round i: } E_{i,active}$$
   $$\text{Competed Targets for Round i: } C_{i,active}$$

   (b) $n_{i,active} < E_{i,active} + C_{i,active}$**:** In this case, there are not enough targets to bind with (and extend on) all encoders and competitors, so they are assumed to bind in a random fashion. This is simulated according to the hypergeometric distribution, as follows:

   $$\text{Code-Extended Targets for Round i } \sim \text{Hypergeometric}((E_{i,active} + C_{i,active}), E_{i,active}, n_{i,active})$$
   $$\text{Competed Targets for Round i } = n_{i,active} - \text{ Code-Extended Targets for Round i,}$$

   where the hypergeometric distribution Hypergeometric(N, K, n) describes the probability, for a population of N total objects with K of type 1 and $N - K$ of type 2, of getting any particular number of successes

of drawing type 1 when making n draws, without replacement, from the population of N total objects. For large values, an approximation is used to generate pseudorandom draws, much as mentioned above for the binomial distribution.

6. **Number of Targets in Next Round.** One computes n_{i+1} as follows. There are two cases, as in the last step, depending on whether the active targets all hybridize:

   (a) $n_{i,active} >= E_{i,active} + C_{i,active}$: one has

   $$n_{i+1} = n_{i,escaped} + n_{i,active} - (E_{i,active} + C_{i,active}) .$$

   (b) $n_{i,active} < E_{i,active} + C_{i,active}$: one has

   $$n_{i+1} = n_{i,escaped}.$$

The process continues through the pre-chosen number of rounds, or until the number of targets (available to hybridize) is zero.

Sampling Step. At this point, one samples, with replacement, from the pool of code-extended targets in order to model the sequencing step that takes place after PCR is performed in an actual experiment. In this step, to imitate how sequencing would act, one pools all of the code-extended targets across all rounds across all species, and then samples with replacement a given number of times, which we naturally refer to as the sequencing depth; this gives the final output to be used in estimation, namely a matrix of counts of how many times each code-extended target is seen by the experimenter in each round.

3.4 Simulation Descriptions and Conditions

We now detail the simulations, and also the parameter choices for them. Each simulation was performed at multiple sequencing depths. For each simulation and for each particular sequencing depth within it, many replications were performed, as described below. Often in the simulations, differing numbers of spike-ins were used for SQUICH and for SRS. For example, using large numbers of spike-ins for SRS will sacrifice performance because sampling bandwidth is used for spike-ins; in contrast, this will improve SQUICH performance because there is better calibration for estimating the number of molecules of a species conditional on the species having observed reads from a specific round. Thus, to be generous to SRS, we reduced the total number of spike-ins used for SRS even when a large number of spike-ins were used for SQUICH.

We note that SRS corresponds to the process typically performed during sequencing.

Simulation 1. Needle in Haystack. The target species were as follows:

$$\begin{array}{l} 20 \text{ species at abundance } 100, \text{ which we call needles,} \\ 1 \text{ species at abundance } 10^x, \text{ for } 12 \leq x \leq 15, \text{ which we call the background, or the haystack.} \end{array}$$

The goal of this simulation is distinct from the others, in that we only wish to test how many of the needles are identified, rather than produce an estimate of the abundances of the species.

Spike-ins were not needed for estimation purposes in this simulation as our goal was simply to determine if we could detect the lower abundance spike-in; however, two spike-ins were used, for programming consistency with the other simulations. (They were set at abundance 100 and 10^x, with the x value in each case the same as the value for the background species.) The spike-ins for SRS were the same.

7 rounds were used and the encoders and competitors were chosen as follows:

$$\begin{aligned} (E_1, E_2, \ldots, E_7) &= (1000, 1000, \ldots, 1000) \\ (C_1, C_2, \ldots, C_7) &= (0, 10^3, 10^4, 10^5, 10^6, 10^7, 10^8). \end{aligned}$$

One simulation was run for each of eight sequencing depths from 10^3 to 10^{10}. Each simulation was performed with 1000 replications.

For each choice of x for the abundance 10^x of the background, there is a corresponding figure, namely Figures E through H for 10^{12}, 10^{13}, 10^{14} and 10^{15}, respectively. In each such figure, the varying colors represent varying sequencing depths, and each point represents a replicate for that sequencing depth. There is of course large overlap of these (1000) points despite the jittering (small numerical deviations that we introduced for display purposes) of the points. Figure H is the same as Figure 2a in the main text, and is retained here for the sake of completeness and for the sake of comparison at the same scaling as the other graphs. We note that in Section 9.1, we also report figures for simulations just as above, with all of the same parameters except for the sequencing depth, which there takes the values 10^{11} through 10^{15}.

Simulation 2. Small Fold Change. A complexity of 2^{18} was used. The target species were as follows:

$$\begin{aligned} &20 \text{ species at abundance } 2000, \\ &(2^{18} - 20) = 262,124 \text{ species at abundance } 1000. \end{aligned}$$

Spike-ins used for SQUICH were 50 species of abundance 2000 and 2 species having each of the abundances 1000, 1500, and 2000. Spike-ins used for SRS were $(1:10) \times 10^{(2:5)}$, i.e. all combinations of multiples of one of $\{1, 2, \ldots, 10\}$ and one of $\{10^2, 10^3, 10^4, 10^5\}$.

One simulation was run for each of four sequencing depths from 10^5 to 10^8. Each simulation was performed with 100 replications. The simulation with a sequencing depth of 10^5 is the particular simulation highlighted in Figure 2b in the main text.

6 rounds were used and the encoders and competitors were chosen as follows:

$$\begin{aligned} (E_1, E_2, \ldots, E_6) &= (100, 100, \ldots, 100) \\ (C_1, C_2, \ldots, C_6) &= (900, 1100, 1300, 1500, 1700, 1900). \end{aligned}$$

The code-extended targets in the first three rounds of SQUICH were omitted, as modeled by replacing encoders 1-3 with a competitor. For programming consistency, we modeled this by removing code-extended targets from rounds 1-3 from sampling.

The simulation results for all four sequencing depths are displayed in Figure M. The top panel is the same as Figure 2b in the main text. As in the last simulation, each color represents a different sequencing depth, and there is one point for each replicate.

Simulation 3. Dynamic Range. A complexity of 2^{12} was used. There were two subclasses of simulations, one for modeling high dynamic range date and one for modeling single cell data.

Target Species and Spike-ins for High Dynamic Range:

$$\begin{aligned} &10 \text{ species of each abundance } (1:10) \times 10^{(0:9)} \\ &(2^{12} - 1045) = 3051 \text{ species at abundance } 100. \\ &\text{Spike-ins: 3 species of each abundance } (1, 5, 10) \times 10^{(5:9)} \end{aligned}$$

The number of the second class of target species was set so that the total number of species sums to 2^{12}. Spike-ins were the same for SQUICH and for SRS.

11 rounds were used and the encoders and competitors were chosen as follows:

$$
\begin{aligned}
(E_1, E_2, \ldots, E_{11}) &= (5, 10, 100, 100, 100, 100, 100, 100, 1000, 1000, 1000) \\
(C_1, C_2, \ldots, C_{11}) &= (0, 10^2, 10^3, 10^4, 10^5, 10^6, 10^7, 10^8, 10^9, 10^{10}, 10^{11}).
\end{aligned}
$$

Simulations were done for the following six sequencing depths: $10^5, 10^6, 10^9, 10^{10}, 10^{11}, 10^{12}$. Each simulation was performed with 100 replications.

The results of the simulation for all sequencing depths are graphed in Figure O; the top panel there is the same as Figure 2c from the main text, except with medians also included and yellow bars for the quantiles included.

Interestingly, the mean is larger than the 99th percentile for SRS in some cases, e.g. for the top right graph. This occurs because the abundance of 100 for the background species is so small relative to the abundance for most of the other species in the simulation and thus the background species is rarely sampled; moreover, the inflation factor r discussed below in Section 3.6 is quite large because some of the (non-background) species have abundance very large relative to the sampling depth. The combined effect of these two issues yields a few very large estimates of the background species with the remaining estimates all 0.

Target Species and Spike-ins for Modeling Single Cell Data:

10 species of each abundance $(1:10) \times 10^{(0:4)}$
100 species of each abundance $(1:10)$
$(2^{12} - 1509) = 2587$ species at abundance 100.
Spike-ins: 3 species of each abundance $(1, 5, 10) \times 10^5$

The number of the second class of target species was set so that the total number of species sums to 2^{12}. Spike-ins were the same for SQUICH and for SRS.

11 rounds were used and the encoders and competitors were chosen as follows:

$$
\begin{aligned}
(E_1, E_2, \ldots, E_{11}) &= (5, 10, 100, 100, 100, 100, 100, 100, 1000, 1000, 1000) \\
(C_1, C_2, \ldots, C_{11}) &= (0, 10^2, 10^3, 10^4, 10^5, 10^6, 10^7, 10^8, 10^9, 10^{10}, 10^{11}).
\end{aligned}
$$

Simulations were done for the following five sequencing depths: $10^5, 10^6, 10^7, 10^8, 10^9$. Each simulation was performed with 10 replications.

The results of the simulation for all sequencing depths are graphed in Figure P.

Choices of Parameters for Hybridization Inefficiency and Thermodynamic Effects for Simulations 1-3. In all three classes of simulations, the probabilities *p.escape* of target escape, *p.encoder.failure* of encoder failure and *p.competitor.failure* of competitor failure, described in Section 3.3, were set to 0.1. As mentioned in Section 3.1, we also ran all three classes of simulations again with all three probabilities set to 0.4.

In all simulations, the discrimination parameter χ described in Section 3.2 was set to 50.

3.5 Statistical Estimators for SQUICH

We now present three estimators that can be used for SQUICH. (Of course other estimation schemes are possible, just as for SRS). Two are general, and one is an estimator modified particularly for use in one of our simulations.

3.5.1 Estimator SEM1

To state the first estimator, which we call $SEM1$, we assume, so that the notation does not get too burdensome, that we are analyzing a SQUICH procedure with d rounds that satisfies

$$\sum_{i=1}^{j}(E_i + C_i) = 10^j$$

for $1 \leq j \leq d$, and $E_i = 10$ for all i, where as above E_i is the number of encoders in the ith round and C_i is the corresponding number of competitors.

We introduce a convenient entity to define the first estimator: for $1 < i \leq d$ let

$$f_i := (\underbrace{1, \ldots, 1}_{i-1}, \underbrace{0, \ldots, 0}_{d-i+1})$$

and let e_i denote the ith unit vector.

After SQUICH, data is generated by sampling with replacement from the pool of code-extended targets, by which recall that we mean the targets that have extended on encoders. These sampled counts, observed from each round, are denoted by $\mathbf{Y} = (Y_1, \ldots, Y_d)$.

Definition 3.1 (SQUICH EstiMator 1 (SEM1) family) *Assume that $E_i > 0$ for $1 \leq i \leq d$. Define*

$$\mathbf{X} = \left(\frac{Y_1}{E_1}, \frac{Y_2}{E_2}, \ldots, \frac{Y_d}{E_d}\right).$$

For $1 < i \leq d$, let r_i be the adjusted residual sum of squares from regressing the vector $\mathbf{X}$ on the two orthogonal predictors e_i and f_i, and define $r_1 = 0$. Also define

$$I = \frac{1}{N} argmax_{1 \leq i \leq d}\, r_i.$$

Define also

$$\overline{X^{(I)}} := \left(\frac{1}{I-1}\sum_{i=1}^{I-1} X_i\right)^{-1} \quad for \quad I > 1.$$

Then the estimate of the original number of targets given by SEM1 is

$$SEM1(\mathbf{X}) = \begin{cases} 1 & for\ I = 1 \\ \sum_{j=1}^{I-1} 10^j + 10^I X_I \overline{X^{(I)}} & for\ I > 1. \end{cases}$$

Note that in practice I is uniquely defined, as r_i is typically minimized at one value with very high probability; but if not, one can define I to be the smallest value of the set of i that minimize r_i.

Intuitively, the SEM1 family uses a disciplined and analytically tractable procedure (regression) to identify the round in which the CGA target sequences are the limiting factor for hybridization rather than the total of competitors and encoders, i.e. the round in which one is in condition (3)(b) of Model 1. The term $\overline{X^{(I)}}$ serves to normalize observed values of X_I.

For intuition, conditional on correctly determining the key round of interest I, the SEM1 estimator estimates the first significant figure by the observation of a random variable sampled with a rate proportional to the number of molecules coded in round i. SEM1 family estimators can control for experimental bias and can be modified to produce unbiased estimates when the quantities E_i vary with i.

Other choices of the normalizing factor $\overline{X^{(I)}}$ are also possible. One straightforward method is to use spike-ins. Denote the abundance of the jth spike-in by z_j. Then we let $z_{i,j}$ be the expected number of molecules of the jth spike-in binding with encoders in round i. Let $Z_{i,j}$ be the counts observed when sampling Z_j in round i. Then one can use

$$\overline{X^{(I)}} := \frac{\sum_j z_{I,j}}{\sum_j Z_{I,j}}$$

For small sampling depth, SEM1 may have poor performance because it fails to select I properly due to high variability in each coding round. In this case other choices of I can be used, including for example the last round where a code-extended target is observed in every round up to and including this round:

$$I := \max\{i \text{ such that } Y_j > 0 \; \forall j \leq i\}.$$

3.5.2 Estimator SEM2

Spike-ins are an integral part of each SQUICH experiment. They are used to normalize counts from samples, but can also be designed to bracket the abundance of the range of values to be measured in a SQUICH experiment. We define an estimator that uses the empirical behavior of spike-ins to quantify unknown molecular abundance.

Definition 3.2 (SQUICH EstiMator 2 (SEM2): Interpolation through spike-ins) *Let A be the set of abundances used for spike-ins, and let S_a denote the set of spike-ins at abundance a.*

Let X_i be the vector of observed sequencing counts of code-extended targets for the ith spike-in. For any target of unknown abundance, let $\mathbf{X}$ be the vector of observed counts of code-extended targets for the target. Then let

$$SEM2(\mathbf{X}) = argmin_{a \in A} \frac{1}{|S_a|} \sum_{X_i \in S_a} ||X_i - \mathbf{X}||_2.$$

In fact, SEM2($\mathbf{X}$) is consistent if there exists $a \in A$ such that the target has abundance a.

3.5.3 Implementation of SQUICH Estimator for Simulation 3

We now describe how SQUICH estimates the abundance of a single species in the modification of SEM1 used for Simulation 3 (dynamic range); we denote the resulting estimate by $\hat{n}$.

Recall that we refer to the key round I as the round in which the target sequences are the limiting factor for hybridization rather than the total of competitors and encoders, i.e. it is the round in which one is in condition (3)(b) of Model 1. In this method, the key round I is identified using the approach described for the definition of SEM1, i.e., with regression.

For a particular target, we define $\mathbf{X} = (X_1, \ldots, X_d)$ to be the vector of observed counts of code-extended targets for that target over all rounds. We calculate $\hat{n}$ in two parts:

$$\hat{n} = \sum_{i=1}^{I-1} \hat{n}_i + \hat{n}_I, \tag{2}$$

by analogy with the definition of SEM1, where $\hat{n}_i$ is the estimated number of targets depleted in round i, i.e. the number in the round that extended on encoders or competitors. We use an estimate of each $\hat{n}_i$ that corrects for: the probability of target escape, the probability of encoder or competitor failure, the fact that the number of encoders E_i varies, and for thermodynamic effects.

Spike-ins are used to compute a normalization factor analogous to the quantity $\overline{X^{(I)}}$ described in $SEM1$, which we describe here. For round i and spike-in s, let $\exp_{is}$ be the number of code-extended targets that would be expected

in round i in the simulation of SQUICH; this is determined by going through the simulation procedure and using expected values when randomization takes place. (Recall that the true original numbers of spike-ins are known.) We denote the number of samples observed for spike-in s in round i (i.e. the number of observed samples after sampling the underlying molecular pool after the SQUICH procedure) by obs_{is}.

Simulations use the term

$$\overline{X^{(I)}} = \frac{\sum_s \exp_{Is}}{\sum_s \text{obs}_{Is}}.$$

In other words, the estimate used for the number of code-extended targets for the round, based on the number X_I of observed samples, is:

$$X_I \overline{X^{(I)}}. \tag{3}$$

Intuitively, multiplication of observed samples for any species in round I by $\overline{X^{(I)}}$ adjusts the number of observed sampled molecules in round I to arrive at an estimate of the underlying number of code-extended targets for that round.

By analogy with the equations in steps (3) and (4) of Section 3.3, define

$$\widehat{E}_{I,active} = E_I(1 - p.encoder.failure) \quad \text{and} \quad \widehat{C}_{I,active} = C_I(1 - p.competitor.failure)$$

The expected value of the hypergeometric probability distribution

$$\text{Hypergeometric}\left(\left(\widehat{E}_{I,active} + \widehat{C}_{I,active}\right), \widehat{E}_{I,active}, n\right),$$

arising as in step (5)(b) in Section 3.3, is $n \dfrac{\widehat{E}_{I,active}}{\widehat{E}_{I,active} + \widehat{C}_{I,active}}$. Therefore, because we are in condition (3)(b) of Model 1 (see Section 1), following just as with the logic in the description of Round 4 of Example 1.1, we expect that if the number of targets that extend on encoders in the key round I is m, then a reasonable estimate of the number of targets available to hybridize at the start of the key round is $m \dfrac{\widehat{E}_{I,active} + \widehat{C}_{I,active}}{\widehat{E}_{I,active}}$. So, combining with the estimate for m given in equation (3), and multiplying by $\dfrac{1}{1 - p.escape}$ to account for the target escape described in step (1) in Section 3.3, the estimate $\widehat{n}_I$ used for the number of targets available to hybridize at the start of the key round I is:

$$\hat{n}_I = X_I \overline{X^{(I)}} \frac{\widehat{E}_{I,active} + \widehat{C}_{I,active}}{\widehat{E}_{I,active}} \frac{1}{1 - p.escape};$$

The estimates $\hat{n}_i$ for the the number of target molecules depleted in earlier rounds are given, for $1 \leq i \leq I - 1$, by:

$$\hat{n}_i := \left(1 + \sum_{i=1}^{k} \binom{n}{i} \chi^{-i}\right) (E_i(1 - p.encoder.failure) + C_i(1 - p.competitor.failure)),$$

where $k = 5$ is used in simulations, as noted in Section 3.2. Then these last two expressions can be combined in equation (2) to arrive at a final estimate of abundance.

3.6 Normalization of SRS Models

To normalize counts in SRS simulations, i.e. to infer an estimate of the number of molecules present from the number of samples, we use the fact that the actual quantities of the spike-ins are known; these values provide an inflation factor similar to the one used when making estimates from SQUICH results. So we use the conventional estimator based on spike-ins, which is given by

$$r := \frac{\sum_{s \in S} \text{actual}_s}{\sum_{s \in S} \text{obs}_s}$$

where S indexes the spike-ins used, obs_s is the observed count of samples of the sth spike-in and actual_s is the (known) abundance of the sth spike-in. Then to estimate abundance for a given target species, the number of observed counts for each species is multiplied by this inflation factor r to provide the estimate of abundance.

3.7 Estimators Used

1. For Simulation 1 ("Needle in haystack"), the same criterion for detection was applied to SQUICH and SRS: if a sequence s_i was sampled, it was considered to be detected.
2. For Simulation 2 ("Small fold change"), which models a background species of abundance 1000 and an above background species of 2000, SEM2 was first used to estimate the abundance of the SQUICH sample. The same criterion for detection was applied for both SQUICH and SRS: if the estimated abundance exceeded 1700, the sequence was said to be above background and was said to be 0 otherwise.
3. For Simulation 3 ("Dynamic range"), a modification of SEM1 (described in Section 3.5.3) was used to estimate the abundance of the SQUICH sample.

In all three simulation types, standard quantification for SRS based on spike-in normalization, as described in Section 3.6, was used.

4 Experimental Methods

In this section, we describe the experimental details of our proof-of-principle demonstration of the SQUICH procedure. This procedure was performed on a set of synthesized oligonucleotides with names prefixed by JSON. We ordered a set of oligonucleotides using degenerate bases in 18 positions where C or G could be present, meaning that each specific oligo name represents a mixture of 2^{18} different specific sequences. Thus, our experiment is on a library of complexity 2^{18}, or $262,144$, sequences. Synthesized oligos were used so that the absolute abundance of each molecule was controlled and performance/estimation of ground truth could be evaluated. We emphasize that this is a model for an experiment where the abundance of each species is sought to be measured without a priori knowledge.

15 experiments were performed. Experiments 1-6 (CH52.01-CH52.06) are SQUICH. Experiments CH53.01-CH53.06 and CH52.07-CH52.09 model conventional sequencing. Experiments 1-3 (CH52.01-CH52.03) were performed with identical conditions and encoder oligonucleotides were added in each round. Experiments 4-6 (CH52.03-CH52.06) were performed with identical conditions but received competitors instead of the encoders in the first round.

SQUICH Encoding steps. Oligonucleotides are labeled JSON# (Table A). A target pool containing the degenerate target library JSON342, and also the individual oligonucleotides JSON343-JSON348 added above background, was prepared. In the encoding experiments, the final concentrations were 600 pM of JSON342, 180 pM of JSON348 and JSON347, 18 pM of JSON346, 1.8 pM of JSON345 and JSON344, and 180 fM of JSON343. The encoding/competition extension reactions were performed in the presence of 20 mM Tris-HCl (pH 8.0), 50 mM KCl, 50 nM of each dNTP (NEB), 12.5 nM dideoxy-CTP (GE Healthcare), 1.5 mM MgCl2 (NEB) and 1.25 units of Native Taq Polymerase (Invitrogen). The initial volume was 50 μL.

Round 1. For experiments 1-3, 1 μL of the encoding oligo, JSON350, was added to 49 μL of the target/enzyme solution to a final concentration of 37.5 pM. For experiments 4-6, 1 μL of the competitor oligonucleotide, JSON454, was added to a final concentration of 37.5 pM. The reaction mixtures were then subjected to the following thermocycler program: 95°C for 5 min, followed by five cycles of 65°C for 30 s and 72°C for 30 s, followed by a 5 min hold at 72°C, and then 4°C.

Name	DNA sequence	Description
JSON342	GCGTTATCGAGGTCSASSASASASSAASASASSASASSASSASSA	Target
JSON343	GCGTTATCGAGGTCCACCACACACCAACACACCACACGACGACGA	Spike-in
JSON344	GCGTTATCGAGGTCCAGGACACACCAACACAGGACAGCACGACGA	Spike-in
JSON345	GCGTTATCGAGGTCCACGAGAGAGCAACACACCACACCACGAGGA	Spike-in
JSON346	GCGTTATCGAGGTCCAGGACACACCAACACAGGAGACGACGACGA	Spike-in
JSON347	GCGTTATCGAGGTCCACCACACACCAAGAGAGGAGAGCACGACGA	Spike-in
JSON348	GCGTTATCGAGGTCGAGGAGAGAGGAAGAGAGGAGACCACGAGGA	Spike-in
JSON437	GCGTTATCGAGGTCGACCAGAGAGGAACAGAGGAGACCACGAGCA	Spike-in
JSON438	GCGTTATCGAGGTCGACGACACACGAACACACGACACGACGACGA	Spike-in
JSON439	GCGTTATCGAGGTCGACCACACACCAACACACGACAGCACGACGA	Spike-in
JSON440	GCGTTATCGAGGTCGACGAGACACGAACAGACGAGACCACGAGCA	Spike-in
JSON441	GCGTTATCGAGGTCGACGAGAGACGAACAGAGGAGACCACGAGCA	Spike-in
JSON442	GCGTTATCGAGGTCGACGAGAGAGCAACACACCACACCACGAGGA	Spike-in
JSON443	GCGTTATCGAGGTCGAGCACACAGCAAGAGACGAGAGCACGACGA	Spike-in
JSON444	GCGTTATCGAGGTCGAGGACACAGCAACACAGGAGACGACGACCA	Spike-in
JSON454	GGGGTSSTSSTSSTSTSSTSTSTTSSTSTSTSSTS - PO4	Competitor
JSON350	isoC-GGGGGTGCTCTTCCGATCTHHHHATATAAATSSTSST SSTSTSSTSTSTTSSTSTSTSSTS-PO4	Code 1 Oligo
JSON351	isoC-GGGGGTGCTCTTCCGATCTHHHHTTACTTATSSTSST SSTSTSSTSTSTTSSTSTSTSSTS-PO4	Code 2 Oligo
JSON352	isoC-GGGGGTGCTCTTCCGATCTHHHHAATCATATSSTSST SSTSTSSTSTSTTSSTSTSTSSTS-PO4	Code 3 Oligo
JSON353	isoC-GGGGGTGCTCTTCCGATCTHHHHTTCATATTSSTSST SSTSTSSTSTSTTSSTSTSTSSTS-PO4	Code 4 Oligo
JSON354	isoC-GGGGGTGCTCTTCCGATCTHHHHACTTAATATSSTSS TSSTSTSSTSTSTTSSTSTSTSSTS-PO4	Code 5 Oligo
JSON355	isoC-GGGGGTGCTCTTCCGATCTHHHHAATATTCATSSTSS TSSTSTSSTSTSTTSSTSTSTSSTS-PO4	Code 6 Oligo
030117_i5_Custom _Tag_adapter	AATGATACGGCGACCACCGAGATCTACACCCTACACGAGCG TTATCGAGGTC	Read 1 PCR primer
030117_Custom _read1_primer	CCACCGAGATCTACACCCTACACGAGCGTTATCGAGGTC	Custom R1 sequencing primer
Barcode Reverse Primer	CAAGCAGAAGACGGCATACGAGAT(barcode)GTGACTGG AGTTCA GACGTGTGCTCTTCCGATC	Index Primer, full length

Table A: The JSON oligonucleotides listed here were synthesized by the Protein and Nucleic Acid Facility (Stanford, CA). All other oligonucleotides were synthesized by Integrated DNA Technologies (Coralville, IA). The sequences for the Read 1 PCR primer, the Custom R1 sequencing primer, and the Barcode oligonucleotides are derived from [4].

Round 2. 1 μL of the second encoding oligonucleotide, JSON351, was added to all 6 reactions to a final concentration of 75 pM. One μL of the competitor, JSON454, was added to all reactions to a final concentration of 600 pM. The reactions were then subjected to thermocycling with the same program as Round 1. This completes Round 2 of the procedure.

Rounds 3-6. 1 μL of an additional encoder (JSON352-355) and 1 μL of competitor were added at each step, followed by the thermocycling program described above. Each encoder was added to a final concentration of 75 pM each. The concentration of added competitor increased by 10-fold during each step from Round 2 to Round 6 (600pM, 6 nM, 60 nM, 600 nM, 6 μM final). The cumulative concentration of competitor was over 6.7 μM. Adjustments were made to compensate for the increasing volume of the reactions during the procedure.

SRS experiments. Experiments CH53.01-CH53.06 model conventional sequencing. An equimolar mixture of the six encoding oligonucleotides, JSON350 to JSON355, was added simultaneously to the target pool reaction mixture to a final collective concentration of 600 pM (100 pM each). This mixture was subjected to the following PCR program: 95°C for 5 min followed by five cycles 65°C for 30 sec and 72°C for 30 sec; followed by a 5 min hold at 72°C and then 4°C. We also performed another model of conventional sequencing in CH52.07-CH52.09 by performing the rounds as in SQUICH but omitting the competitors, to be conservative in case the performance of rounds benefited conventional sequencing. These experiments had worse performance than conventional sequencing in CH53.01-CH53.06 and were omitted from analysis.

PCR. For some experiments, PCR was done in duplicate (denoted A and B in S3 Table) and sequenced reads from each experiment were pooled. The sequencing barcodes were added using standard protocols and sequenced using custom primers (gifts from the Jin Billy Li lab; see Table B). The 50 μL reaction mixtures were composed of 25 μL of the SQUICH reaction with 1X Phusion HF buffer (NEB), 200 μM dNTPs, 0.5 μM of each primer, and 1 unit of Phusion DNA Polymerase (NEB). We used the following PCR program: 98°C for 2 min; four cycles of 98°C for 10 s, 55°C for 10 s, and 72°C for 30 s; nine cycles of 98°C for 10 s and 72°C for 30 s; and 72°C for 5 min. The PCR products were then purified via AmpureXP magnetic beads (Beckman Coulter) at a ratio of 1:1.8. The purified products were quantified on the QUBIT fluorometer and pooled. The final pool was quantified by the Stanford Functional Genomics Facility using the Agilent Bioanalyzer (DNA High Sensitivity).

DNA sequencing. DNA sequencing was performed on an Illumina MiSeq instrument (San Diego, CA) by the Stanford Functional Genomics Facility using the MiSeq Reagent Nano Kit v2 (300-cycles), Paired End setting, 60 bases per cycle. Data parsing was performed using custom scripts to identify reads containing targets, assign codes, and perform statistical analysis as described in detail in Section 5.

5 Analysis of Experimental Data

Experimental data was generated blindly, that is, the estimation procedure was chosen before the experiment was performed, and all tests in this sequencing run are reported. Parsing was performed by scanning each sequencing read for a target code matching our designed target code libraries, and identifying the sequence encoding the round the respective encoder was added appearing adjacent to the target code. We required that both R1 and R2 of the sequencing reads match the same target code and also that the encoder sequences matched in R1 and R2. Parsed data is given in S3 Table. Note that we refer to spike-ins in this experiment as molecules where ground truth is known to the experimenter. These spike-ins were not used for normalization, at least not in the same way as they were in the simulations; this will be discussed below. Two technical replicates of SQUICH were pooled (to increase power) and analyzed with a modified SEM1 estimator.

In previous experiments, we found that target escape, encoder failure and competitor failure suggest that only about 60% of molecules are depleted from the pool each round. Thus in our analysis, we incorporate this by assuming for the sake of estimation that 6^i molecules are depleted in each round rather than 10^i. In more detail, we arrive at the estimate 6^i as follows: 8000 is the fold increase of the highest spike-in that is completely depleted by round 6 (and which we assume has known abundance), and 5 is the number of rounds required to deplete it from detection with an encoder (i.e. no code-extended target for round 6 is observed for this oligo). So we estimated the effective fold depletion $f = 6$, instead of the the theoretical $10-$fold depletion, using the following formula to correct for

BC241	CGTAATGA
BC242	TTGCTTAG
BC243	TCTTGTTC
BC244	GTGGCTTC
BC245	TGTTCGAT
BC246	TCATTCAG
BC247	GTGGAGAG
BC248	GTAGAAGT
BC249	TGGAGCAT
BC250	GAAGGAGA
BC251	CGAATGTA
BC252	TCGTGAAT
BC253	GAATAGCT
BC254	TTGTCACA
BC255	CTGGAGGC
BC256	TGTCAGCT
BC257	GTTCTTCG
BC258	TTACACGT
BC259	GTAGCCAG
BC260	TGAGAAGG
BC261	CCATATGA
BC262	CGATCCTA
BC263	TGACTAGC
BC264	TAACTCTG

Table B: Sequencing adapter barcode oligonucleotides were a kind gift from the laboratory of Jin Billy Li at Stanford University. See [4].

Fold	code1	code2	code3	code4	code5	code6	SEM_A (Proportional estimate of Fold)
79954.9	37	38	56	17	37	14	971827.749
79954.9	165	181	184	45	56	8	834187.28
7996.4	24	37	30	8	7	0	66606.25
800.5	2	5	1	1	1	0	9325.78
81	0	2	2	0	0	0	951.67
1	0	2	2	0	0	0	951.67
800.5	1	5	1	0	0	0	621.48
1	0	2	1	0	0	0	512.24
1	1	1	1	0	0	0	475.83

Table C: Example of SQUICH data parsing and estimation.

experimental inefficiencies in hybridization:

$$f = 10^{\log_{10}(8000)/5} \approx 6.$$

We define the variant of the SEM1 estimator we will use in the following way. First, for a fixed sequence, let code_i be the number of codes from the ith round sequenced, i.e. the number of code-extended targets from the ith round sequenced. Also define dig_i to be the indicator that codes from the sequence were observed from all rounds 1 through round i, inclusive. Additionally, define exc_dig_i (for "exclusive digit") to be the indicator that $\text{dig}_i = 1$ but $\text{dig}_{i+1} = 0$. Then we define

$$\begin{aligned} SEM_A \quad &= \quad \text{code}_1 * f + (\text{code}_2 * f^2) * (\text{exc\_dig}_2) + \sum_{i=3}^{5} \left(\text{code}_{i-1} f^{i-1} + \text{code}_i f^i\right) * (\text{exc\_dig}_i) \\ &\quad + \left(f^5 * \text{code}_5 + \text{code}_6 * f^6\right) * \text{dig}_6. \end{aligned}$$

SEM_A may be 0; for example this would occur if exactly 1 count of each of code 2 and code 5 is observed and no others are observed. Then we finally define our variant $\widetilde{SEM}1$ of SEM1 as

$$\widetilde{SEM}1 = \begin{cases} SEM_A & \text{if } SEM_A \neq 0 \\ \sum_{i=1}^{6} \text{code}_i & \text{if } SEM_A = 0. \end{cases}$$

For control libraries, i.e. the libraries for experiments $CH52.01 - CH52.09$, which do not have competitors, the species abundance was estimated as the total number of counts observed for each sequence.

We then computed Spearman (rank) and Pearson correlations between estimated abundances and the ground truth values. As noted in the main text, these correlations for SQUICH exceeded those for conventional sequencing despite lower depths of sequencing. See Figure 3c from main text for Pearson correlations and Figure C here for Spearman correlations. However, note that Spearman correlations are not very meaningful given that the ground truth abundance is 1 for all but six of the species. In fact, perhaps unintuitively, correlation decreases as sequencing depth increases; the first six species are estimated in the right order, but the many remaining species are estimated to be in varying orders because the number of species that are actually sampled at least one time increases, whereas the abundances of all of these should be estimated as being the same.

To estimate the fold reduction enabled by SQUICH compared to conventional libraries, we use a quantity that we call effective sequencing depth (ESD); it estimates fold reduction while providing a way of normalizing for variation in sampling depth. Given a measure of experimental performance, ESD is an estimate of the depth required by a theoretical SRS experiment (i.e. one without noise introduced during sequencing) to achieve, on average, performance comparable to that of the experiment.

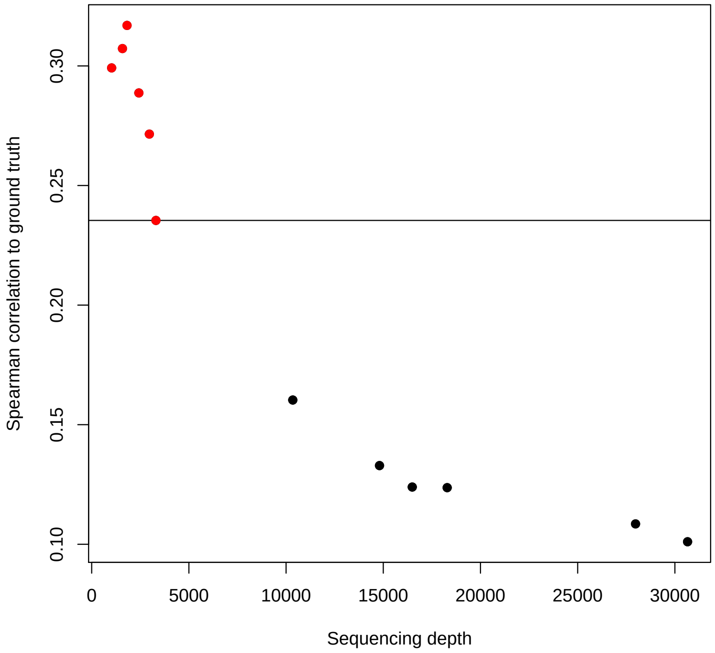

Figure C: Plot of Spearman (rank) correlations against sequencing depth, for SQUICH (red) and for conventional sequencing (black).

For the calculation of ESD, we compute a metric M_1, which is defined as

$$M_1 := \sum_{i=1}^{6} \pi_i \sigma_i,$$

where σ_i is the rank of the ith spike-in based on the empirical rank of SEM1 (for SQUICH) or on total counts (for conventional sequencing) and π_i is the rank of the (known) concentration of the ith spike-in.

To calculate ESD, given an experimental depth of d_0 reads, for

$$\alpha = .2, .25, .3, .35, .4, .45, .5, .55, .6, .65, .7, .75 \text{ and } \alpha = 2, 3, 4, 5, 6, 7, \tag{4}$$

we simulated 1000 replications of SRS at depth αd_0 and reported the fraction of replicates of SRS at depth αd_0 that exceeded the observed value of M_1. This fraction is an empirical p-value for M_1 under the null hypothesis that the experiment performs as well as a theoretical SRS experiment at depth αd_0.

For experiment i, the empirical p-value for a fixed α is denoted as $\hat{p}_i^{(\alpha)}$. If the SQUICH (respectively, conventional sequencing) experiments with experimental depth d_0 were performing equivalently to a theoretical SRS experiment with αd_0 samples, the set of $\hat{p}_i^{(\alpha)}$ over the set of SQUICH (respectively, conventional sequencing) experiments would be distributed as uniform $(0, 1)$. For each α, we computed the Kolmogorov-Smirnov goodness of fit test statistic between the uniform distribution $U(0, 1)$ and either (i) the set of values $\{\hat{p}_i^{(\alpha)}\}$ over all the SQUICH experiments indexed by i, or (ii) the set of values $\{\hat{p}_i^{(\alpha)}\}$ over all the SRS experiments indexed by i. (As discussed in Section 4, only experiments CH53.01-CH53.06 were used for SRS; however, including CH52.07-CH52.09 as well in this yields very similar results.) Call the value of these test statistics $X(\alpha)$. Then we define ESD, for each of SQUICH or SRS, as the value of α giving the best fit, or, formally,

$$ESD := \text{argmin}_\alpha X(\alpha).$$

Computed p values are in Table D. Note that experiments CH52.07, CH52.08, CH52.09 are listed here, but we omitted them from the analysis because they were conventional sequencing experiments that performed worse than the conventional sequencing experiments modeled in the CH53 series.

There are 18 values of α for which we computed $X(\alpha)$, as presented in (4). Let j, varying from 1 to 18, index these values α_j, where $\alpha_1 = .2$, $\alpha_2 = .25$, up to $\alpha_{18} = 7$. Then, in Figure D, we plot $X(\alpha_j)$ against j.

SQUICH did much better than SRS: its $p-$values are most uniform (i.e. $X(\alpha)$ is smallest) when we move the ESD to large values of α, i.e. when we compare SQUICH to theoretical SRS experiments performed at much higher depths. On the other hand, the actual SRS experiments are most uniform when compared to theoretical SRS experiments performed at a fraction of the experimental depth, namely $\alpha \approx .5$, which is the value of α for the index 7. Roughly speaking, this tells us that "empirical SRS" is as accurate as a theoretically perfect SRS experiment with only about 50% of the sampling depth of empirical SRS; SQUICH is like a theoretically perfect SRS experiment with 5 to 7 times more samples than were used in SQUICH.

Pooled, the six SQUICH replicates had an ESD of about 5, meaning to achieve the same precision, 5 fold fewer samples are required compared to the number expected with SRS without experimental noise. The ESD of conventional sequencing was $\approx .5$, meaning ≈ 2 times more samples than predicted to be needed by SRS are required to overcome experimental noise. Taking the ratio of SQUICH ESD to conventional ESD gives the estimate that SQUICH achieves a 10x depth reduction compared to conventional sequencing.

Our experiments are conservative in comparison with conventional sequencing. Our experimental model of conventional sequencing is in a regime where encoders that allow targets to be extended onto library molecules that can be included in a sequencing library were limiting, which attenuates the dynamic range usually seen in SRS and increases performance. Second, our experiments are with unpurified oligos and using degenerate bases (Table A), meaning any target sequence has other target sequences 1 mismatch away.

Experiments outside of the scope of the paper will address optimizing the performance of the SQUICH system: this protocol has not yet been optimized, and hence we expect even greater fold reductions in required sequencing with optimization, and oligosynthesis error, pipetting errors or sequencing bias and will be studied in the future.

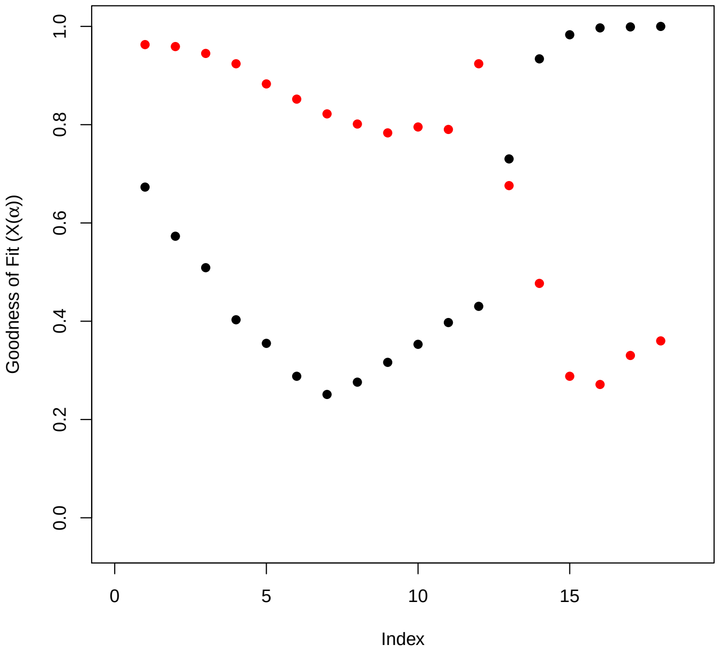

Figure D: $X(\alpha)$ as a function of the index of values of α, for SQUICH (red) and for conventional sequencing (black).

Experiment	CH52.01	CH52.02	CH52.03	CH52.04	CH52.05	CH52.06	CH52.07	CH52.08	CH52.09	CH53.01	CH53.02	CH53.03	CH53.04	CH53.05	CH53.06
Starting Depth	1818	2427	3305	1583	2967	1026	54714	57213	55598	10345	27977	30653	14806	16490	18287
α															
0.2	0.000	0.001	0.037	0.003	0.001	0.003	0.590	0.632	0.616	0.046	0.273	0.327	0.075	0.108	0.146
0.25	0.000	0.000	0.041	0.000	0.003	0.008	0.736	0.722	0.712	0.071	0.340	0.427	0.172	0.173	0.222
0.3	0.001	0.002	0.055	0.007	0.008	0.011	0.803	0.824	0.812	0.120	0.476	0.491	0.203	0.237	0.293
0.35	0.000	0.001	0.076	0.008	0.004	0.005	0.841	0.739	0.869	0.133	0.565	0.597	0.229	0.288	0.348
0.4	0.000	0.004	0.117	0.015	0.009	0.008	0.780	0.792	0.812	0.181	0.630	0.645	0.317	0.353	0.366
0.45	0.003	0.006	0.148	0.019	0.011	0.013	0.833	0.843	0.842	0.222	0.677	0.712	0.317	0.428	0.432
0.5	0.010	0.008	0.178	0.021	0.013	0.022	0.868	0.903	0.889	0.251	0.736	0.780	0.389	0.476	0.511
0.55	0.003	0.011	0.228	0.032	0.019	0.017	0.932	0.926	0.928	0.276	0.803	0.813	0.405	0.501	0.561
0.6	0.004	0.018	0.227	0.050	0.032	0.014	0.945	0.955	0.943	0.312	0.802	0.871	0.483	0.550	0.583
0.65	0.014	0.026	0.264	0.038	0.018	0.017	0.962	0.950	0.961	0.353	0.855	0.869	0.518	0.600	0.636
0.7	0.006	0.043	0.211	0.006	0.038	0.011	0.971	0.981	0.976	0.397	0.863	0.774	0.564	0.644	0.659
0.75	0.010	0.031	0.076	0.011	0.064	0.027	0.978	0.991	0.983	0.421	0.897	0.802	0.597	0.675	0.728
2	0.130	0.241	0.324	0.124	0.309	0.040	1.000	1.000	1.000	0.715	1.000	0.999	0.897	0.938	0.972
3	0.285	0.393	0.523	0.227	0.489	0.097	1.000	1.000	1.000	0.934	1.000	1.000	0.988	0.998	1.000
4	0.379	0.556	0.712	0.322	0.594	0.159	1.000	1.000	1.000	0.983	1.000	1.000	0.999	0.999	0.999
5	0.514	0.619	0.807	0.438	0.528	0.263	1.000	1.000	1.000	0.997	1.000	1.000	1.000	1.000	1.000
6	0.555	0.497	0.892	0.513	0.628	0.297	1.000	1.000	1.000	0.999	1.000	1.000	1.000	1.000	1.000
7	0.428	0.611	0.924	0.609	0.738	0.360	1.000	1.000	1.000	1.000	1.000	1.000	1.000	1.000	1.000

Table D: Empirical p-values for SQUICH for various values of α.

6 Applications for Single Cell Sequencing

In this setting, simple probes hybridize to RNA (or in other applications, protein or DNA) sequences to transform each to a unique target sequence such as a CGA sequence. These CGA sequences are then used as the target sequences for input to SQUICH. Each probe consists of a region that hybridizes to a specific RNA sequence, and a second region that is a CGA sequence. Total RNA is incubated with probes, and the target CGA sequences of these probes bound to RNA are eluted as input to SQUICH (Fig A). The same principle can be applied to measure protein abundance using antibody-coupled oligos.

As an example, we described our simulations of SQUICH performance on pooled single cell RNA-seq in Section 3.4. The way that this would work in practice is as follows. Targets are broken apart into sub-sequences, and the full length target encodes two pieces of information:

$$\underbrace{A\,C/G\,A\,C/G\,A\ldots\,C/G\,A}_{\text{CGA ``part'' from molecule 1}}\;\underbrace{A\,C/G\,A\,C/G\,A\ldots\,C/G\,A}_{\text{CGA ``part'' from molecule 2}}\,.$$

In the simulation, "part 1" encodes the barcode for a cell, and "part 2" the gene (Fig Aa). (The "parts" could also be tails of two ligation probes, subsequently recombined by ligation to a single target, creating a quadratic number of targets from the "parts" (Fig Ab).)

7 Statistical Model and Proof of Claim

7.1 Statistical Model

We now present a statistical model that allows us to formally compare SQUICH with SRS.

Suppose that one starts with a set of molecules in a tube. There are k species of molecules, and the abundance of the molecules of species s is denoted by n_s. These abundances are unknown to the experimenter. We consider two sampling frameworks. To compare the methods, we will define them and when their precision is compared, we define a "fair comparison" as a sampling regime with equal numbers of draws from each method; note that in the SQUICH case, we perform operations on the molecules before the draws, but the costs of these are minor compared to the costs from sampling when sequencing.

1) **Simple Random Sampling (SRS),** with replacement, is formally defined as follows. We make m total draws from the original tube containing all of the molecules. For any given realization of the draws, the number of draws for species s is denoted by h_s, so that one has $h_1 + h_2 + \ldots + h_k = m$. Thus these random variables follow a multinomial distribution, namely

$$(h_1, h_2, \ldots, h_k) \sim \text{Multinomial with parameters } \mathbf{p} \text{ and } m,$$

where

$$\mathbf{p} = \left(\frac{n_1}{\sum_{s=1}^{k} n_s}, \frac{n_2}{\sum_{s=1}^{k} n_s}, \ldots, \frac{n_k}{\sum_{s=1}^{k} n_s} \right).$$

2) **SQUICH.** We first implement the rounds of the SQUICH procedure, which may modify the molecules at each round. We sample from a subset of the modified molecules at the end of the procedure. The procedure is as described in items (5) (Modeling of Hybridization) and (6) (Number of Targets in Next Round) in Section 3.3. We omit the sources of error from items (1)-(4) for the sake of this simple model, as our goal here is to demonstrate the benefits of SQUICH rather than analyzing the detailed statistical properties of the completely specified particular experimental procedure, which introduces unique sources of noise. We demonstrated the benefits of SQUICH even when incorporating the sources of error using the simulations and physical experiments.

We start with n_s molecules of each species. In each round, we implement the procedure, introducing E_{si} encoders and C_{si} competitors in the ith round for each species s. In each round, the number of code-extended targets for round i and the number of competed targets for round i depends on whether one is in the case (a) or (b) of item (5) in Section 3.3.

Then a realization of the random variables over all rounds $i = 1, \ldots, I$ is a set of I vectors

$$(n_{si}, z_{si}),$$

where n_{si} and z_{si} are determined by the procedure described below. Their meanings, given for the sake of following the intuition of the procedure, are, for rounds $1 \leq i \leq I$ and species $1 \leq s \leq k$,

$$z_{si} = \text{ the number of code-extended targets for species } s \text{ in the } i^{th} \text{ round,}$$

and

$$\begin{aligned} n_{si} \quad = \quad & \text{the number of molecules of species } s \text{ available in round } i \\ & \text{to bind with (and extend on) encoders and competitors.} \end{aligned}$$

For round 1, we define

$$n_{s1} = n_s.$$

Now suppose that we are in round i, with $1 \leq i \leq I$. Then, for each species s, the realization z_{si} is determined as follows:

Case (a): $n_{si} \geq E_{si} + C_{si}$. Then

$$z_{si} = E_{si}.$$

Case (b): $n_{si} < E_{si} + C_{si}$. Then

$$z_{si} \sim \text{Hypergeometric}((E_{si} + C_{si}), E_{si}, n_{si})$$

where the hypergeometric distribution $\text{Hypergeometric}(N, K, n)$ describes the probability, for a population of N total objects with K of type 1 and $N - K$ of type 2, of getting a particular number of type 1 when making n draws, without replacement, from the population of N total objects.

Finally, for each species s, for rounds $i < I$, the quantity $n_{s(i+1)}$ is determined by the following:

In Case (a), one has

$$n_{s(i+1)} = n_{si} - (z_{si} + C_{si}).$$

In Case (b), one has

$$n_{s(i+1)} = 0.$$

This completely describes the random vectors

$$(n_{s1}, z_{s1}), (n_{s2}, z_{s2}), \ldots, (n_{sI}, z_{sI}).$$

Even in the more complicated setting with errors, these can be determined recursively as random variables conditional on the random variables in the previous round.

7.2 Proof of Claim

Claim 7.1 *We assume the setting of SQUICH without hybridization inefficiency and without thermodynamic effects (see Sections 3.1 and 3.2), so that essentially we abstract away from that type of experimental noise. Suppose the abundance of two species are respectively* $x_1 10^{y_1}$ *and* $x_2 10^{y_2}$ *with* $x_1, x_2 \in \{1, 2, \ldots, 9\}$ *and non-negative integers*

$y_1 < y_2$. Fix $0 < p < 1$. Then, when using constant amounts of encoders $E_i = 10$, and using total number of encoders and competitors cumulatively through the ith round set equal to 10^i, SQUICH needs only

$$\left(\frac{y_2+1}{y_1}\right)\log(1/p)$$

samples to achieve a probability of detection of $1-p$, whereas simple random sampling (SRS) requires at least

$$\left(10^{y_2-y_1-1}\right)\log(1/p)$$

samples.

Proof 7.2 *We start with the proof for the part of the claim related to SQUICH. For $s = 1, 2$ and $1 \le i \le y_2 + 1$, define z_{si} to be the number of code-extended targets for species s in the tube in the ith round. Then, for $s = 1, 2$, one has*

$$\begin{aligned} z_{si} &= 10 && \text{if} && i \le y_s \\ 0 \le z_{si} &\le 10 && \text{if} && i = y_s + 1 \\ z_{si} &= 0 && \text{if} && i > y_s + 1 \end{aligned} \tag{5}$$

Now consider the process of simple random sampling from all of the code-extended targets that are pooled together in the sampling tube. (5) gives the number of code-extended targets in the ith round, and thus the probability q_1 of sampling, in one draw, an element of species 1 is

$$q_1 = \frac{10y_1 + z_{1(y_1+1)}}{10y_2 + z_{2(y_2+1)}}.$$

One has

$$q_1 \ge \frac{10y_1}{10y_2+10} = \frac{y_1}{y_2+1}. \tag{6}$$

Recall consider the simple random sampling step of SQUICH. As the sampling is with replacement, the probability of not sampling any molecules of species 1 in m draws (i.e. $P(Bin(m, q_1) = 0)$) is $(1-q_1)^m$. Using the fact that $1 + x \le e^x$ for all x (e.g. because $f(x) = e^x$ is a convex function with tangent line $y = 1 + x$ at $x = 0$) and using (6), one has

$$P(Bin(m, q_1) = 0) = (1-q_1)^m \le e^{-mq_1} \le e^{-\frac{my_1}{(y_2+1)}}$$

For $m = \frac{y_2+1}{y_1}\log\left(\frac{1}{p}\right)$, the right hand side is equal to $e^{-log(1/p)} = p$, and thus the left hand side is less than or equal to p for any value of m at least that large, completing the proof (for the claim regarding SQUICH).

Proof for SRS. *A similar result for the probability of failing to sample species 1 with simple random sampling is a classical result following from the Poisson approximation of the binomial when m is large and p is small. However, we give an elementary proof for completeness.*

The probability of sampling a molecule of species 2 in one draw is

$$\frac{x_2 10^{y_2}}{x_1 10^{y_1} + x_2 10^{y_2}}.$$

Therefore the probability of sampling at least one molecule of species 1 in m draws is simply given by

$$1 - \left(\frac{x_2 10^{y_2}}{x_1 10^{y_1} + x_2 10^{y_2}}\right)^m.$$

Now this quantity is greater than or equal to $1-p$ *only when* m *is large enough, so that the following chain of equivalent conditions holds:*

$$1-\left(\frac{x_2 10^{y_2}}{x_1 10^{y_1}+x_2 10^{y_2}}\right)^m \geq 1-p$$

$$\Longleftrightarrow$$

$$\left(\frac{x_2 10^{y_2}}{x_1 10^{y_1}+x_2 10^{y_2}}\right)^m \leq p$$

$$\Longleftrightarrow$$

$$\left(\frac{x_1 10^{y_1}+x_2 10^{y_2}}{x_2 10^{y_2}}\right)^m \geq \frac{1}{p}$$

$$\Longleftrightarrow$$

$$m\log\left(\frac{x_1 10^{y_1}+x_2 10^{y_2}}{x_2 10^{y_2}}\right) \geq \log\left(\frac{1}{p}\right)$$

$$\Longleftrightarrow$$

$$m \geq \frac{\log\left(\frac{1}{p}\right)}{\log\left(1+\frac{x_1 10^{y_1}}{x_2 10^{y_2}}\right)}. \tag{7}$$

Now the inequality $log(1+x) \leq x$ *holds for* $x > -1$*, as can be seen, e.g., by taking logs of both sides of the inequality* $1+x \leq e^x$ *used above, as* $\log y$ *is an increasing function. Thus one has*

$$\log\left(1+\frac{x_1 10^{y_1}}{x_2 10^{y_2}}\right) \leq \frac{x_1 10^{y_1}}{x_2 10^{y_2}},$$

and therefore

$$\frac{1}{\log\left(1+\frac{x_1 10^{y_1}}{x_2 10^{y_2}}\right)} \geq \frac{x_2 10^{y_2}}{x_1 10^{y_1}} \geq 10^{y_2-y_1-1}.$$

Thus if m *satisfies the bound (7), then it must also satisfy the bound*

$$m \geq 10^{y_2-y_1-1}\log\left(\frac{1}{p}\right),$$

i.e., at least that many draws are required to ensure a probability of detection of $1-p$*.*

8 Generality of SQUICH

We use the concept of a multiset, a generalized version of a set in which each element can have some multiplicity attached. A "target sequence pool" is a multiset of molecules (here, oligonucleotides) whose underlying abundance is the vector of parameters of interest. An encoder is a molecule that, when a target hybridizes with and extends on it, results in tagging the target with information that encodes which encoder hybridized to the target. A competitor is a molecule that, when a target hybridizes with and extends on it, results in failure of the target to be sequenced.

The general conditions for SQUICH are defined as follows:

Definition 8.1 (Conditions for SQUICH: Competitor and Encoder multisets) *The target, competitor and encoder multisets are multisets (here molecules, and more specifically oligos) with the following properties:*

1. *There is an ordering of encoders and competitors such that each target molecule binds to each encoder and competitor set with decreasing preference according to this ordering. For sequential SQUICH, this order is determined by sequential addition of encoders and competitors in each "round".*

2. *For each set in the ordering, for each target that could exist in the pool, there is a multiset of encoders and competitors (which could be the empty set) such that for each target, the probability distribution of its binding each encoder and competitor can be predicted (which could be, for example, the uniform distribution).*

The paradigm for SQUICH is very general. Ordering can be controlled through sequential additions of molecules or by reaction conditions such as melting temperature (TM) of encoders or competitors if performed in one pot. A special case of SQUICH is, of course, traditional sampling where the number of encoders E_1 in round 1 is effectively infinite and all other E_i and C_i are zero. SQUICH enables arbitrary choices of E_i and C_i, changing the entropic composition of sampled molecules to reparameterize the sampling distribution.

In a molecular biological experiment implementing the SQUICH procedure, many variables are available for application-specific optimality: the temperature, the concentration of each oligo, including potential use of modified bases, concentration of enzymes, buffers, nucleotides, extension times and many other variables that can determine the empirical performance. Designing variable concentrations of each competitor and/or encoding sequence will allow each target set to be measured in its own base representation. Time or other variables such as temperature can control order of target binding to each encoder or competitor for one-pot SQUICH procedures.

We describe SQUICH for nucleic acids for the sake of exposition and because our experiments and simulations to model noise are based on this system. However, SQUICH is more general and can be applied to arbitrary sets of sequences and molecules. For example, one could also do the following:

- Targets can be endogenous sequences or any engineered set of library molecules.
- Targets and encoder/competitor libraries need not be in one-to-one sequence relationships. For example, targets could be more or less complex than the set of encoders or competitors.
- Each sequence in each library (target, competitor, encoder) can vary in abundance in each round.
- Functions such as weighted averages, that is inner products, can be computed by varying temperature, including wobble or universal bases such as Inosine in encoder and competitor sequences.

We note that SQUICH can even be thought of as an example of a still more general paradigm, in which one performs what are in essence carefully designed computations with molecules before sampling with sequencing. Moreover, we are hopeful that this paradigm can be applied in ways that will enable greater efficiencies in many areas. Here are some potential ways the paradigm could be used:

- Sequential occupancy of one set of molecules by another such that the round at which molecules occupied increases information content of the ultimate assay. An example is sequential hybridization of a set of tagged reverse transcription primers so that primers tagging molecules in late rounds encode information about the abundance of the original molecules.
- Targets, in the nomenclature of SQUICH, can be reprobed multiple times by blocking the ends of the targets. Probe molecules, called encoders in the nomenclature of SQUICH, can bind and extend on the targets multiple times, including in the presence of (sequential) competitor pools. In this case, the set of targets will be measured indirectly through measurement of probe molecules that are sequenced.
- Targets can be endogenous molecules, probed multiple times as above. In this case, the probes extend on endogenous sequences and can encode partial information (through sequence complementarity in the process of polymerase extension).

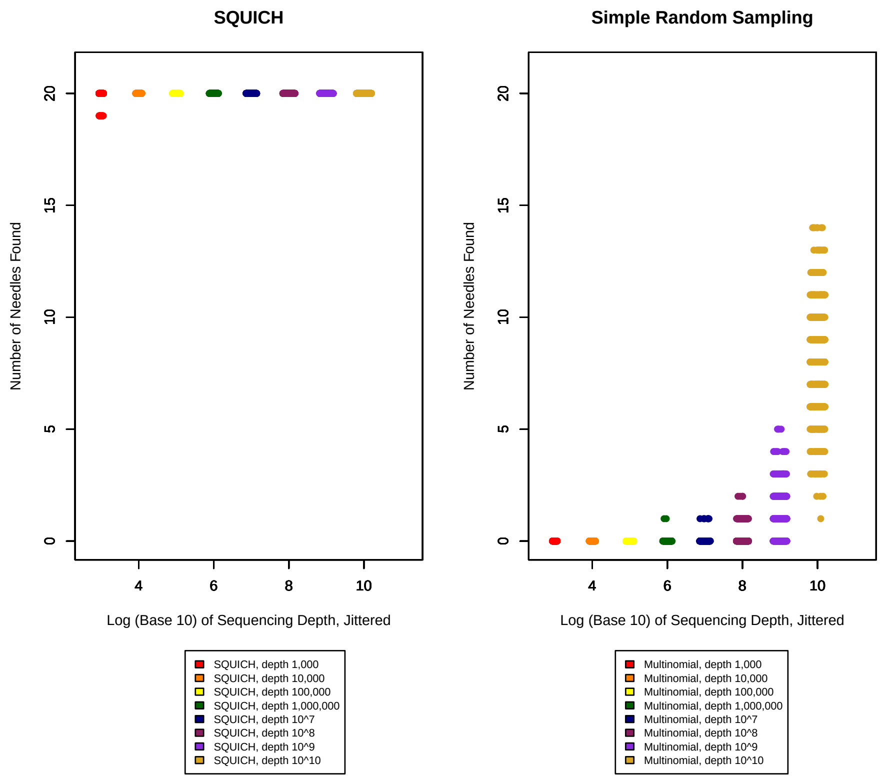

Figure E: Simulation 1. Haystack abundance 10^{12}. Sequencing Depths 10^3 to 10^{10}

9 Supplemental Figures and Discussion for Simulations

9.1 Simulation 1: Needle in Haystack

Performance of SQUICH vs. SRS with 1000 simulations at a variety of sampling depths shows that SQUICH enables improved detection. The figures show the number of "needles" (the 20 species at abundance 100) detected within the background (a species of abundance 10^x, where x varies from 12 to 15 depending on the simulation), at various sequencing depths. Figures E through H show the graphs for each of the four abundances 10^{12} through 10^{15}; each figure reports the number of targets detected for the various sequencing depths 10^3 through 10^{10}. Figure H is the same as Figure 2a in the main text, and is shown here for ease of comparison. In each figure, each color represents a different sequencing depth, and there is one dot for each replicate, with of course large overlap of these (1000) dots despite the jittering (introduced small deviations) of the dots. We then present four more figures, Figures I through L, which show simulations for the same four abundances as in the previous four figures, and with all of the same parameters except for the sequencing depth, which takes the values 10^{11} through 10^{15}.

Table E shows, for each abundance (including abundances not shown in these figures), the number of samples required by SRS or SQUICH to find all 20 needles.

As mentioned in Section 3.1, we also ran all of the simulations again with all three probabilities of hybridization inefficiency set to 0.4, and for Simulation 1, the plots from the simulations with probabilities set to 0.4 all look very similar to the original results with probabilities set to 0.1, and thus the plots are not presented here.

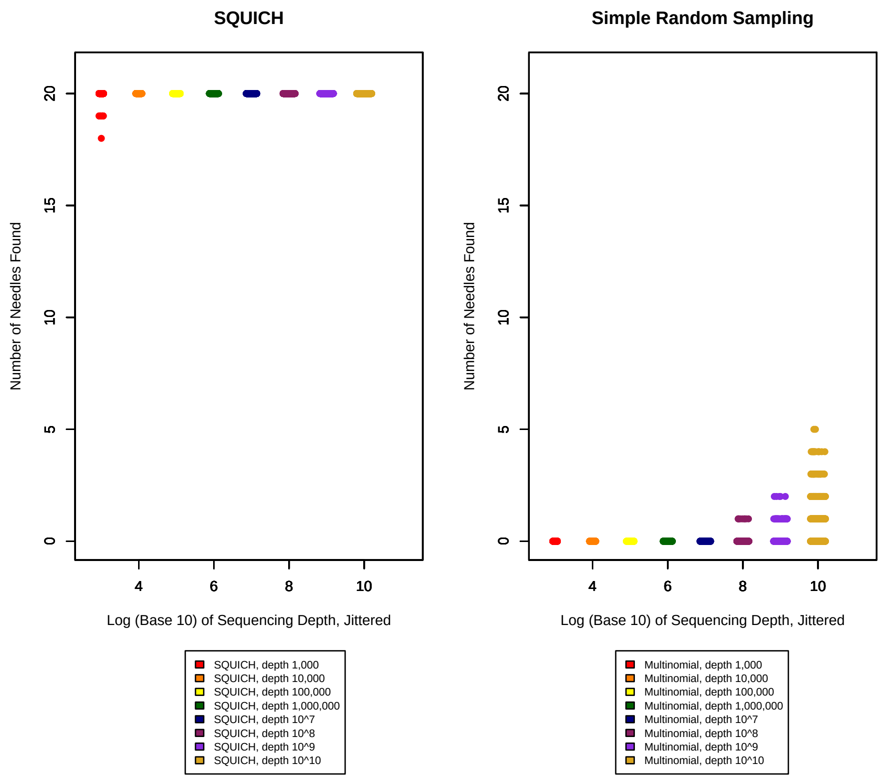

Figure F: Simulation 1. Haystack abundance 10^{13}. Sequencing Depths 10^3 to 10^{10}

Abundance	Number of Samples Needed for SRS	Number of Samples Needed for SQUICH
10^5	10^5	2000
10^6	10^6	2000
10^7	10^7	2000
10^8	10^8	2000
10^9	10^9	2000
10^{10}	10^{10}	2000
10^{11}	10^{11}	2000
10^{12}	10^{12}	2000
10^{13}	10^{13}	2000
10^{14}	10^{14}	2000
10^{15}	10^{15}	2000

Table E: Number of samples required by SRS or SQUICH to find all 20 needles, for each abundance (including abundances not shown in the figures).

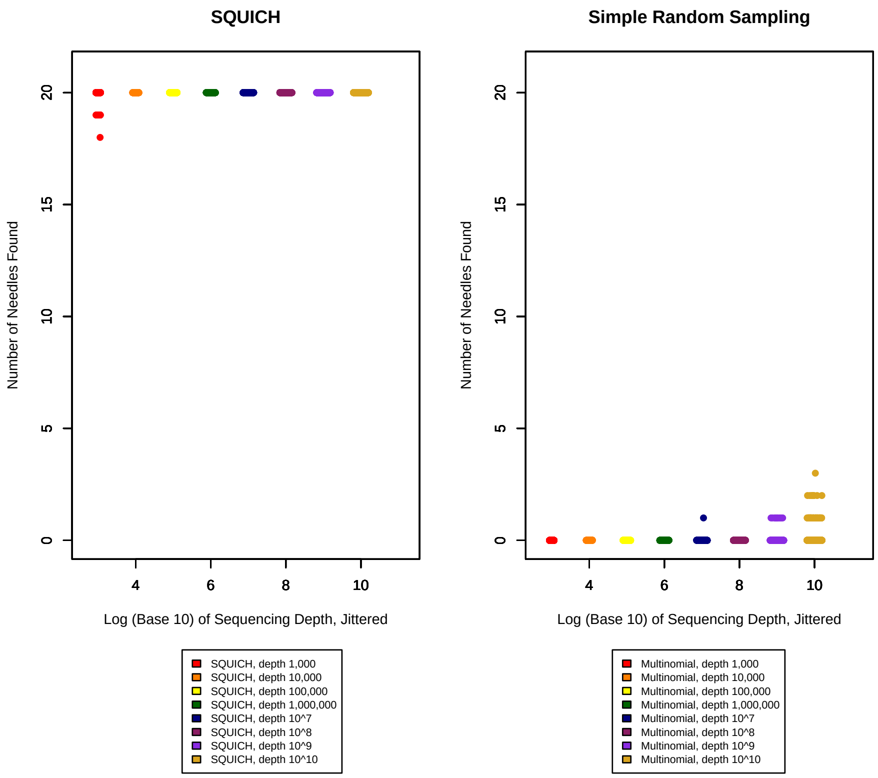

Figure G: Simulation 1. Haystack abundance 10^{14}. Sequencing Depths 10^3 to 10^{10}

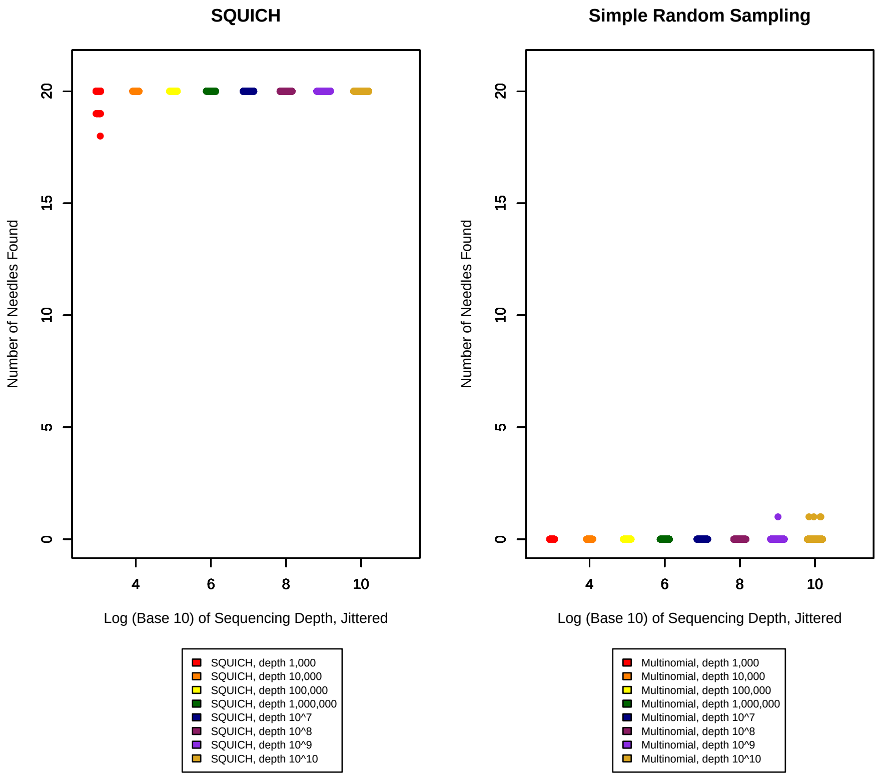

Figure H: Simulation 1. Haystack abundance 10^{15}. Sequencing Depths 10^3 to 10^{10}. This is the needle in the haystack simulation shown in Figure 2a in the main text.

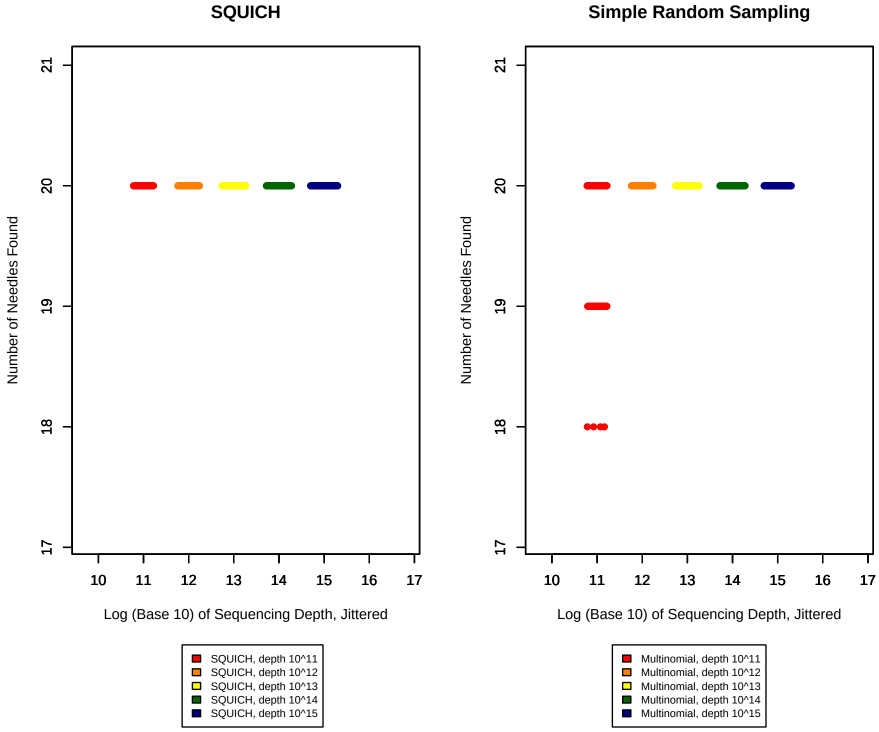

Figure I: Simulation 1. Haystack abundance 10^{12}. Sequencing Depths 10^{11} to 10^{15}

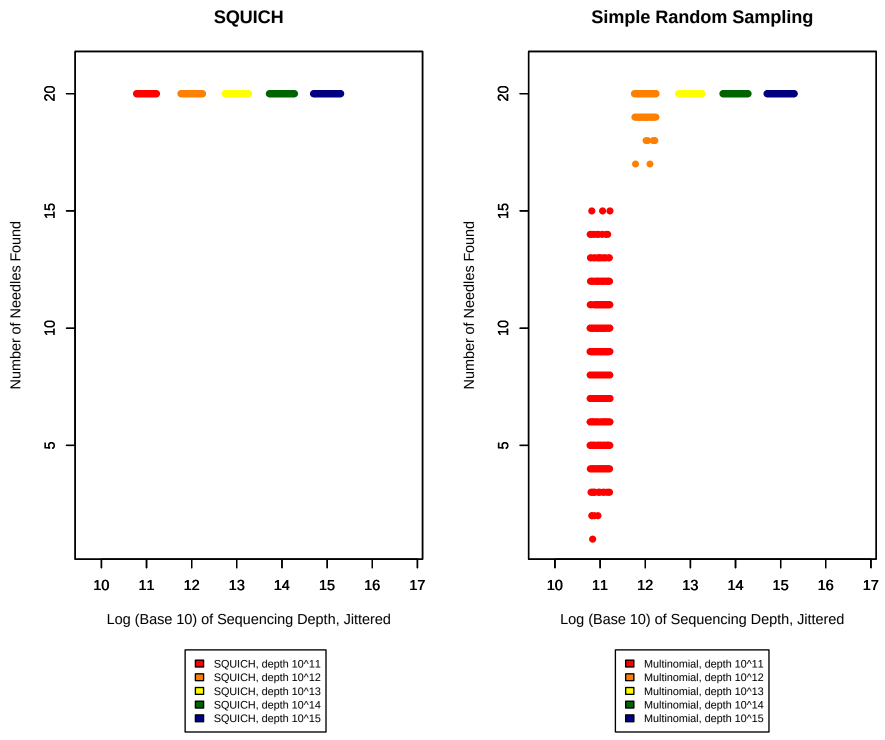

Figure J: Simulation 1. Haystack abundance 10^{13}. Sequencing Depths 10^{11} to 10^{15}

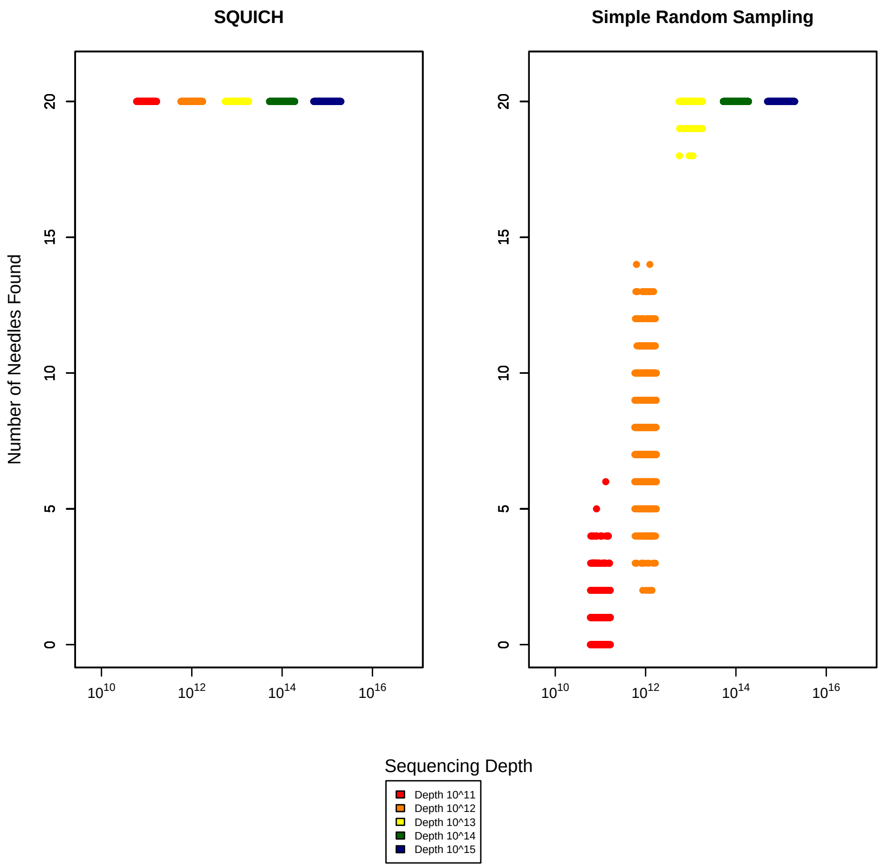

Figure K: Simulation 1. Haystack abundance 10^{14}. Sequencing Depths 10^{11} to 10^{15}

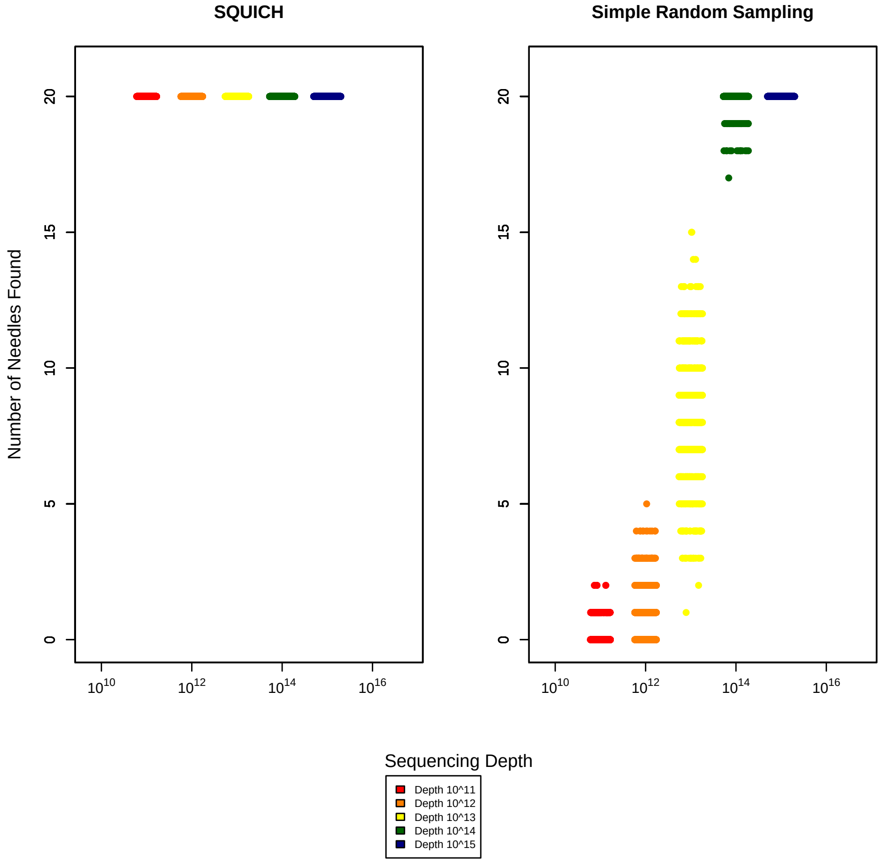

Figure L: Simulation 1. Haystack abundance 10^{15}. Sequencing Depths 10^{11} to 10^{15}

9.2 Simulation 2: Small Fold Change

Simulations for modeling small fold change are reported in Figure M. The top panel here is the same as Figure 2b in the main text, and the other panels are simulations run under the same conditions but with different sequencing depths.

As mentioned in Section 3.1, we also ran the simulation again with all three probabilities of hybridization inefficiency set to 0.4 (rather than 0.1). The corresponding figure does differ visibly from Figure M, so we report it in Figure N, noting that the relative strength of the results for SQUICH as compared to SRS is not as large, but the results are still more favorable for SQUICH than for SRS.

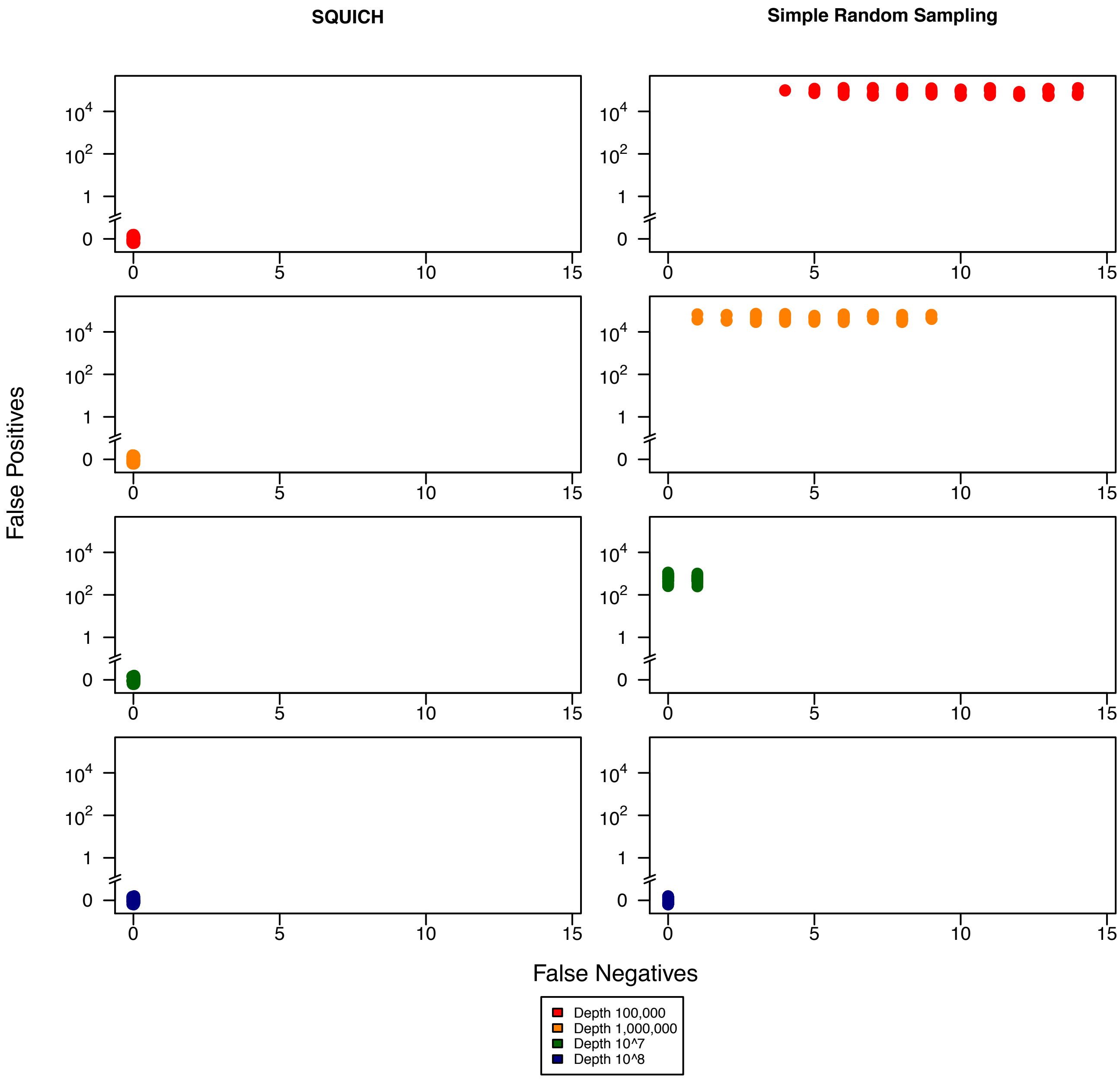

Figure M: Simulation 2. Multiple sequencing depths for conditions of Figure 2b. SQUICH enables detection of small fold changes, here 2 fold in 20 species, in a background of $2^{18} - 20 = 262,124$ samples. All true positives are recovered with 0 false positives. To achieve the same performance with SRS, 10^3-fold more samples are required. The $y-$axis is $\log_{10}$-scale, and jittering is performed for both x and y values. The top panel here is the same as Figure 2b in the main text, and the other panels display simulations run under the same conditions but with other sequencing depths.

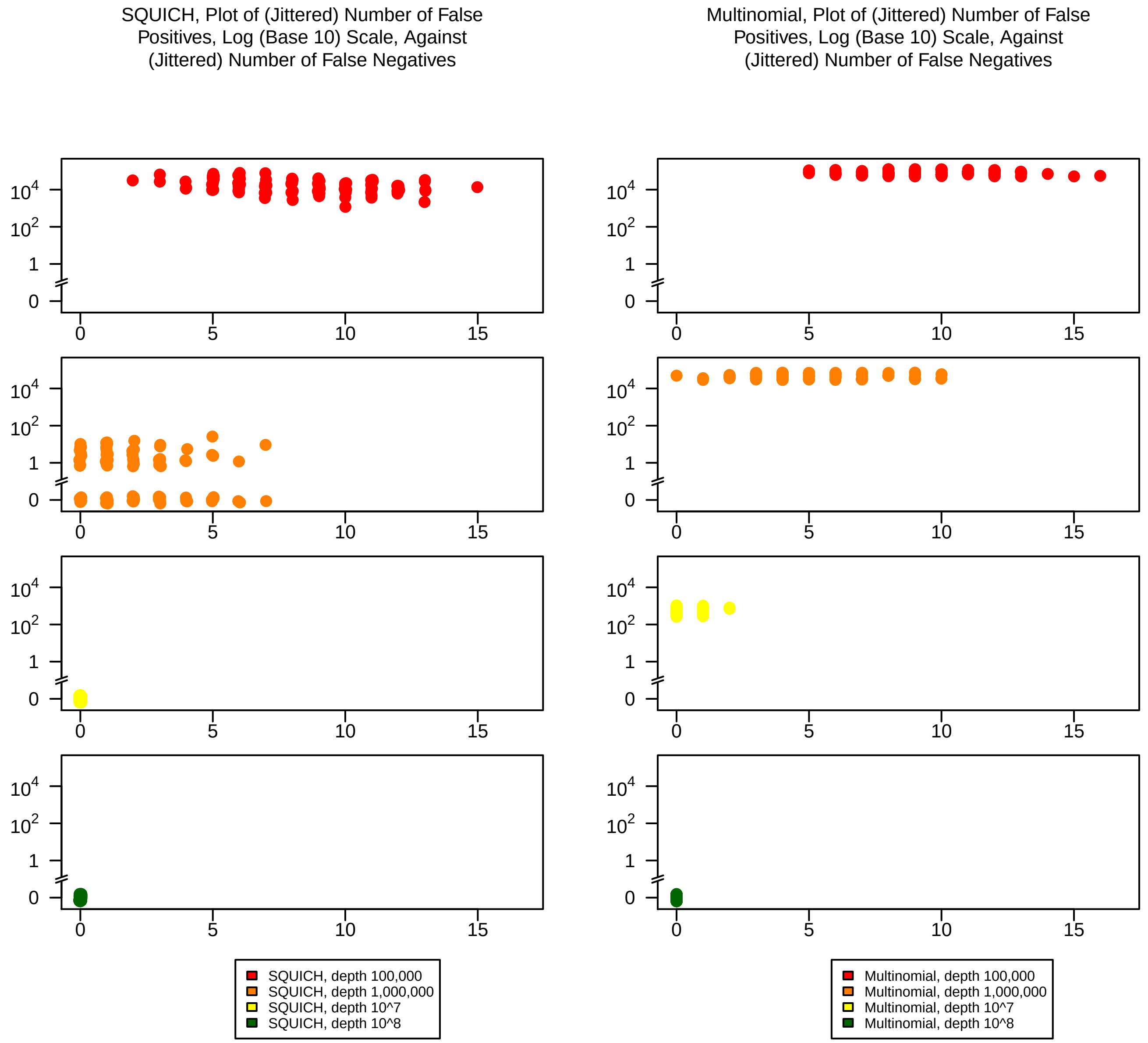

Figure N: Simulation 2. Multiple sequencing depths for conditions of Figure 2b, except with the three probabilities of hybridization inefficiency set to 0.4 rather than 0.1. SQUICH enables detection of small fold changes, here 2 fold in 20 species, in a background of $2^{18} - 20 = 262,124$ samples. The $y-$axis is $\log_{10}$-scale, and jittering is performed for both x and y values.

9.3 Simulation 3: Dynamic Range

In primary biological samples such as microbial DNA and digital protein quantification, quantification of all species varying over up to 9 or more orders of magnitude to detect two-fold changes in any species is required. We modeled this with 2^{12} species of abundance taking values $a \times 10^b$ with $1 \leq a \leq 10$ and $0 \leq b \leq 9$ (Fig O).

SRS at $100,000$ samples has poor performance at quantifying species of the first 4-5 orders of magnitude (see Fig O); the maximum counts observed in the background species coincide with the expected value of species with ground truth of about 10^3 fold higher. With SRS, the background and the first three orders of magnitude are indistinguishable; SQUICH also improves erroneous estimation of background due to multiple testing (Fig O). At depth 10^5, SQUICH has better estimation performance as measured by average L_2 log-loss, a measure of fold change precision, than SRS at depth 5×10^9 (S2 Table).

The results of the simulation for all sequencing depths for modeling single cell sequencing are graphed in Figure P.

As mentioned in Section 3.1, we also ran both classes of simulations again with all three probabilities set to 0.4 (rather than 0.1). For both types of simulation in Simulation 3, the plots look very similar to the original results, and thus are not presented here.

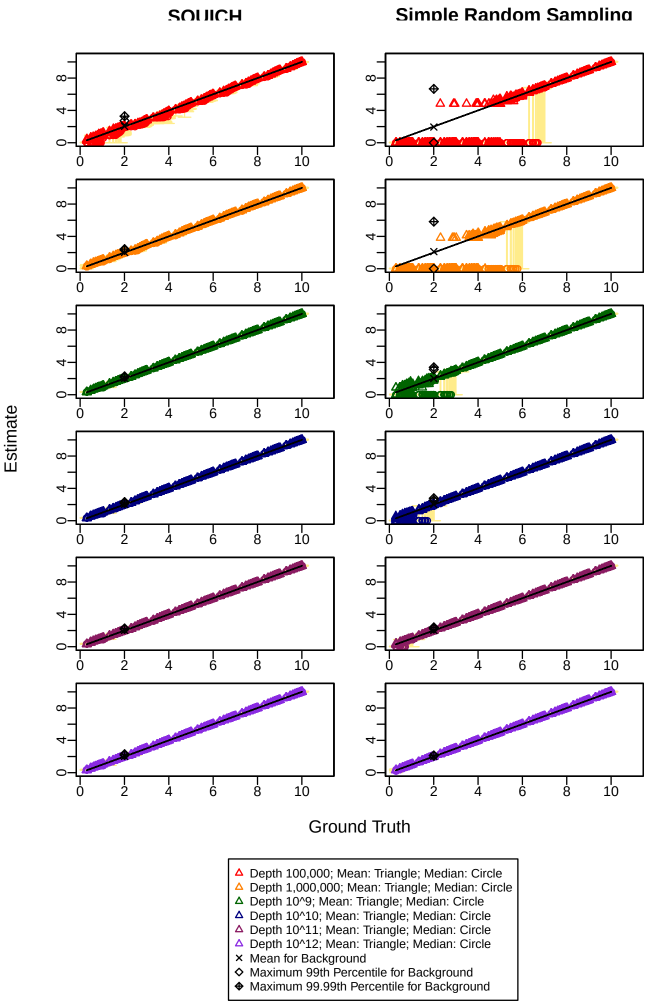

Figure O: Simulation 3. Multiple sequencing depths for conditions for high dynamic range example discussed in paper. SQUICH enables detection of small and large molecular abundances across a dynamic range of 9 orders of magnitude with 10^5 samples (SQUICH at left). SRS at this depth (right) has higher dropout with poor performance in detecting species of the first 4 orders of magnitude, requiring 10^{11} reads to have comparable performance. The x- and y-axes are $\log_{10}$-scale and are jittered; the axes are labeled by the logs of the values. Yellow bars denote the 25-75th quantiles of measurement for SQUICH and SRS. SQUICH gives a savings of $> 10^6$ fold. The top panel shows the same data as Figure 2c from the main text, except with medians also included and yellow bars for the quantiles included.

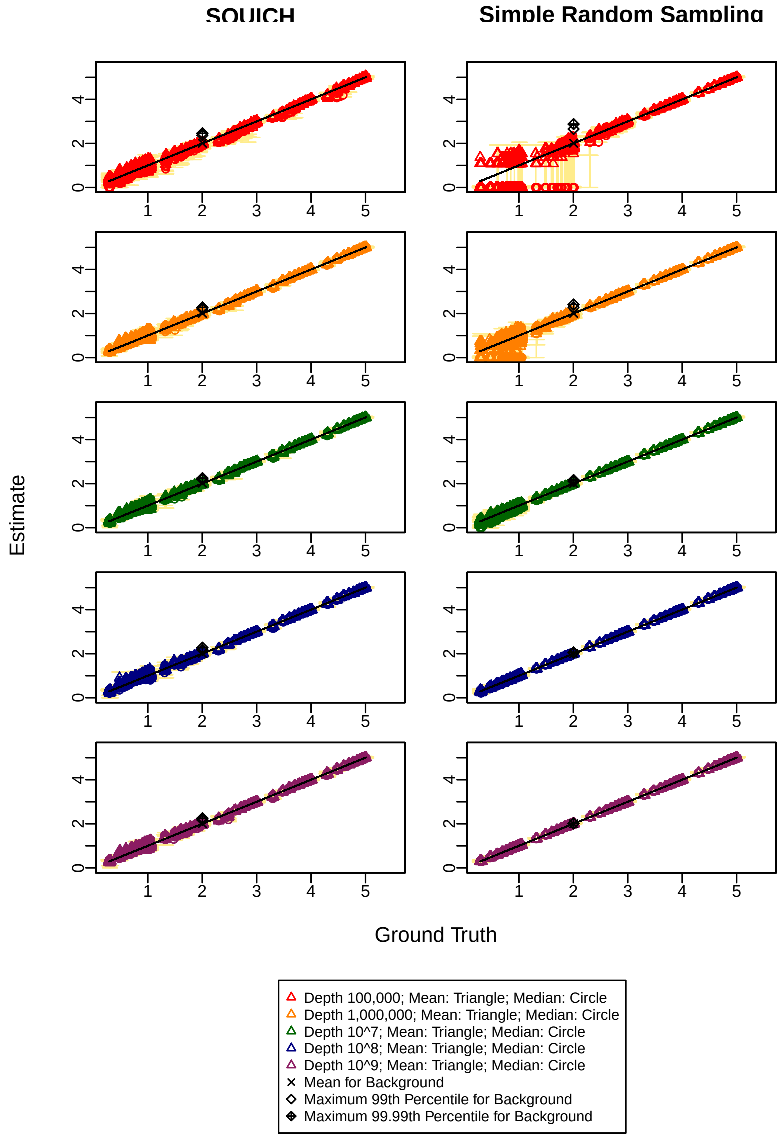

Figure P: Simulation 3. SQUICH enables detection of small and large molecular abundances across a dynamic range of 5 orders of magnitude (SQUICH at left), modeling single cell sequencing profiles: 10 values each were set to each range in (1:10)x $10^{(0:4)}$ and 100 at each copy number from 1:10; roughly 2600 species were set to a background level of 100. SQUICH has low drop-out at 10^5 reads whereas SRS at this depth (right) has higher dropout with poor performance in detecting species of low abundance requiring 100x more samples than SQUICH to achieve similar performance. The x- and y-axes are $\log_{10}$-scale and are jittered; the axes are labeled by the logs of the values. Yellow bars denote the 25-75th quantiles of measurement for SQUICH and SRS.

References

[A] Zadeh JN, Steenberg CD, Bois JS, Wolfe BR, Pierce MB, Khan AR, Dirks RM, Pierce NA. NUPACK: analysis and design of nucleic acid systems. J Comput Chem. 2011; 32(1):170-173. doi:10.1002/jcc.21596
