# Supplementary material for: Molecular sampling at logarithmic rates for next-generation sequencing
Source: PLoS Comput Biol. 2019 Dec 12;15(12):e1007537. doi: 10.1371/journal.pcbi.1007537 (PMC6932819; doi:10.1371/journal.pcbi.1007537)
Supplement: S2 Table — (PDF) [file pcbi.1007537.s003.pdf]

Supplementary Table S2

Raw R output of analysis of the mean, median, and 1%,5%,95% and 99% quantiles of drop-outs and log2loss computed as ||log(x/y)||_2^2 where y is ground truth and x is estimated value, across 100 simulations. When x=0, we set x=1 to compute the loss. Library complexity was 2^12 for both simulations.

Numerical results for high dynamic range reported in the main text. Spike-ins set to c(1,5,10)x10^(5:9) repeated 3 times.

Drop out rates for 10^6 samples and log MSE are comparable to or better than SRS at 10^(11), a savings of 10^5 fold sampling in high dynamic range simulation.

[1] "SQUISH"

[1] "dropout"

depth	mean	median	.01quantile	.05quantile	.95quantile	.99quantile
1e+05	427.48	429	392.99	396.95	456.05	461.01
1e+06	3.94	4	1.00	2.00	7.00	7.01
1e+09	1.10	1	0.00	0.00	3.00	3.01
1e+10	1.01	1	0.00	0.00	3.00	4.00
1e+11	1.07	1	0.00	0.00	3.00	4.00
1e+12	1.10	1	0.00	0.00	3.00	4.00

[1] "logL2loss"

depth	mean	median	.01quantile	.05quantile	.95quantile	.99quantile
1e+05	159932.1	159816.2	157179.2	157823.9	162362.9	163019.8
1e+06	142791.1	142785.6	142137.0	142311.7	143278.2	143420.3
1e+09	141561.2	141569.2	141246.8	141306.2	141848.3	141915.9
1e+10	141562.7	141556.6	141064.1	141308.1	141814.6	141904.3
1e+11	141550.2	141562.5	141164.3	141296.4	141785.7	141844.6
1e+12	141555.5	141555.8	141127.1	141315.7	141815.3	141865.5

[1] "SRS"

[1] "dropout"

depth	mean	median	.01quantile	.05quantile	.95quantile	.99quantile
1e+05	3706.19	3706.0	3694.94	3697.00	3715.00	3718.01
1e+06	3597.91	3598.0	3584.99	3589.95	3606.05	3609.05
1e+09	2867.46	2867.5	2829.98	2835.95	2895.05	2909.00
1e+10	818.81	820.0	755.98	781.95	858.00	871.00
1e+11	50.26	50.5	39.00	41.95	60.05	62.01
1e+12	2.74	3.0	0.00	1.00	5.00	5.01

[1] "logL2loss"

depth	mean	median	.01quantile	.05quantile	.95quantile	.99quantile
1e+05	298937.6	299011.2	296151.9	297022.0	300750.5	301043.3
1e+06	300619.2	300713.5	296305.0	297076.4	303389.2	305050.9
1e+09	217939.6	217905.0	215879.1	216393.8	219546.5	219819.0
1e+10	163085.0	163085.1	161689.6	161856.8	164268.2	164555.0
1e+11	142501.8	142509.2	141940.8	142048.6	142992.7	143168.6
1e+12	141310.0	141310.6	141162.3	141189.5	141431.3	141446.8

Numerical results for single cell model reported in main text; simulations were repeated 10 times.
(1:10)x(10^(c(0:4)) repeated 10 times; (1:10) each repeated 100 more times.

spikes set to(1,5,10) *10^(5) repeated 3 times.

Drop out rates for 10^5 samples and log MSE are comparable to SRS at 10^7, a savings of 100 fold sampling single cell simulation

[1] "SQUISH"
[1] "dropout"

	mean	median	.01quantile	.05quantile	.95quantile	.99quantile
1e+05	79.33	79	60.98	66.85	91.10	96.04
1e+06	13.14	13	6.99	8.00	19.05	20.00
1e+07	12.29	12	5.99	7.00	18.05	20.01
1e+08	12.63	12	6.00	7.00	19.05	21.03
1e+09	12.28	12	5.00	6.95	18.00	19.03

[1] "logL2loss"

	mean	median	.01quantile	.05quantile	.95quantile	.99quantile
1e+05	12432.56	12409.98	12140.89	12216.39	12675.47	12726.43
1e+06	11664.37	11665.88	11522.58	11553.70	11760.29	11832.13
1e+07	11567.49	11562.32	11463.64	11484.28	11647.66	11679.00
1e+08	11553.25	11557.99	11449.15	11467.36	11633.04	11657.87
1e+09	11561.09	11566.52	11399.15	11468.50	11641.77	11648.53

[1] "SRS"
[1] "dropout"

	mean	median	.01quantile	.05quantile	.95quantile	.99quantile
1e+05	2173.35	2174.5	2116.81	2133.90	2213.20	2225.12
1e+06	700.17	701.0	668.94	671.95	727.00	731.06
1e+07	76.42	75.0	60.98	65.00	92.05	98.03
1e+08	0.00	0.0	0.00	0.00	0.00	0.00
1e+09	0.00	0.0	0.00	0.00	0.00	0.00

[1] "logL2loss"

	mean	median	.01quantile	.05quantile	.95quantile	.99quantile
1e+05	39562.57	39600.33	38487.99	38683.32	40394.12	40841.07
1e+06	13727.92	13731.30	13553.72	13581.22	13848.29	13918.81
1e+07	11454.23	11453.77	11382.62	11404.89	11505.22	11528.36
1e+08	11328.37	11327.37	11279.81	11297.81	11364.16	11371.10
1e+09	11265.39	11265.60	11252.96	11255.24	11274.62	11275.66

There were 50 or more warnings (use warnings() to see the first 50)

Squish 10^5 vs SRS 10^7 loss reported

> 12432.56/11454.23
[1] 1.085412
